# Molecular taxonomy of pancreatic neuroendocrine tumors reveals *BEND2*-fusions-driven transcriptional plasticity and therapeutic vulnerabilities

**DOI:** 10.1016/j.xcrm.2026.102642

**Published:** 2026-03-06

**Authors:** Xiaofan Lu, Philippe Baltzinger, Li Xu, Antonin Fattori, Sehrish Khan Bazai, Fatima Alhourani, Marie-Pierrette Chenard, Philippe Bachellier, Pietro Addeo, Véronique Debien, Clara Vacheret, Alessio Imperiale, Patrick Pessaux, Wenxuan Cheng, Martin Balzinger, Jean-Emmanuel Kurtz, Irwin Davidson, Xiaoping Su, Bernard Goichot, Gabriel G. Malouf

**Affiliations:** 1 Department of Cancer and Functional Genomics, Institute of Genetics and Molecular and Cellular Biology, CNRS/INSERM/UNISTRA, Illkirch, France; 2 Department of Endocrinology, Diabetology and Nutrition, Strasbourg University Hospital, Strasbourg, France; 3 Department of Pathology, Strasbourg University Hospital, Strasbourg, France; 4 Department of Hepato-Pancreato-Biliary Surgery and Liver Transplantation, Strasbourg University Hospital, Strasbourg, France; 5 Early Phase Clinical Trials Unit, Institut Bergonié, Bordeaux, France; 6 Department of Nuclear Medicine, Institut de Cancérologie Strasbourg Europe, Strasbourg, France; 7 Department of Digestive and Endocrine Surgery, Strasbourg University Hospital, Strasbourg, France; 8 Department of Medical Oncology, Strasbourg University Hospital, Hôpital de Hautepierre, Strasbourg, France; 9 Department of Bioinformatics and Computational Biology, The University of Texas MD Anderson Cancer Center, Houston, TX, USA

**Keywords:** pancreatic neuroendocrine tumors, *BEND2* fusions, progenitor cells, transcriptional reprogramming, clinical outcome

## Abstract

Pancreatic neuroendocrine tumors (pNETs) exhibit substantial clinical and molecular heterogeneity. Using bulk and single-nucleus RNA sequencing, we identify five molecular subtypes: Hedgehog-high, Alpha-like, Hypoxia-high, Gastrin-high, and Progenitor-like. The Gastrin-high and Progenitor-like subtypes associate with poor clinical outcomes. *BEND2* gene fusions occur in 5% of pNETs, all belonging to the Gastrin-high subtype, which shows activation of the late endocrine progenitor FEV regulon. Functional studies in pNET cell models demonstrate that *BEND2* fusions drive transcriptional reprogramming, promoting a shift from ASCL1^+^ endocrine states toward neurodevelopmental, mesenchymal, and immune-related gene programs. Single-nucleus analysis reveals complex multicellular ecosystems, with *NOTCH3*-mediated signaling between tumor cells and myofibroblasts emerging as a potential therapeutic vulnerability. Gastrin-high tumors exhibit CD8^+^ T cell infiltration alongside PD-1/PD-L1 upregulation, suggesting potential responsiveness to immune checkpoint blockade. These findings define a molecular taxonomy of pNETs and nominate tumor-intrinsic and microenvironmental programs as actionable targets.

## Highlights

• *BEND2* gene fusions define a clinically aggressive Gastrin-high subtype of pNETs
• *BEND2* fusions are mutually exclusive from *MEN1*, *DAXX*, and *ATRX* mutations
• *BEND2* fusions reprogram neuroendocrine cells toward mesenchymal and inflamed states
• Chromatin and immune programs nominate therapeutic targets in *BEND2*-fusion tumors

## Graphical abstract

**Figure:**
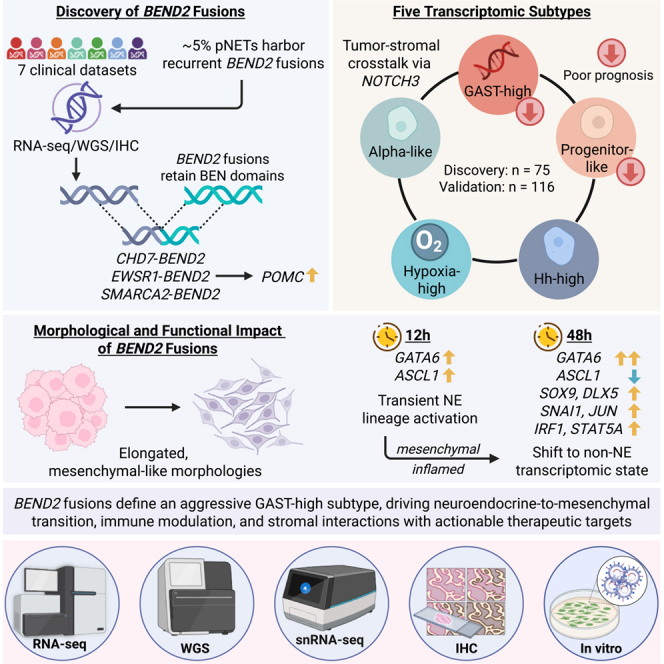

## Teaser

Lu et al. identify *BEND2* fusions in ∼5% of pancreatic neuroendocrine tumors, defining an aggressive Gastrin-high subtype. Integrating bulk and single-nucleus RNA sequencing with functional assays, *BEND2* fusions are shown to drive transcriptional plasticity and nominate chromatin and immune signaling programs as actionable therapeutic targets.

## Introduction

Pancreatic neuroendocrine tumors (pNETs) are the second most common type of pancreatic neoplasms after adenocarcinomas and have shown a rising incidence over the past decade.^1^ They are classified by functional status based on the presence or absence of hormonal syndromes (e.g., hypoglycemia in insulinomas).^2^ While most pNETs are non-functional and often diagnosed incidentally or due to mass effects,^3^ nearly half of patients present with distant metastases at diagnosis, resulting in poor 5-year survival rates.^1^

The World Health Organization (WHO) classification stratifies pNETs into three grades based on differentiation, Ki67 index, and mitotic count, while distinguishing well-differentiated pNETs from poorly differentiated neuroendocrine carcinomas (NECs), which require distinct prognostic and therapeutic considerations.^4^ Despite available treatments—including surgery, somatostatin analogues, and systemic therapies such as everolimus or sunitinib—clinical outcomes remain suboptimal, and current grading and staging systems lack predictive biomarkers to guide precision therapy in this heterogeneous disease.^3^

Genomic studies have identified recurrent mutations in *MEN1*, *DAXX*, and *ATRX*, as well as alterations in the PI3K/AKT/mTOR pathway.^5^ Structural variants affecting tumor suppressors (*MTAP*, *ARID2*, *SMARCA4*, *MLL3*, *CDKN2A*, and *SETD2*) and in-frame gene fusions (*EWSR1-BEND2* and *EWSR1-FLI1*) have also been described,^5^^,^^6^^,^^7^ although their functional and clinical relevance remains incompletely understood.

Transcriptome- and epigenome-based classifications have revealed multiple pNET subtypes, though their interpretation is limited by cohort size and clinical annotation.^7^^,^^8^^,^^9^ The latest proposed subtype classification identified alpha-cell-like and beta-cell-like gene expression profiles, which are associated with high *ARX* expression and *DAXX/ATRX* mutations, respectively. Two other subtypes, stromal/mesenchymal and proliferative, displayed distinct characteristics related to the tumor microenvironment (TME) and included a majority of the NECs in the study.^10^ Recently, a proteogenomic study of 108 non-functional pNETs further identified four proteomic subtypes associated with distinct molecular features, immune infiltration patterns, and therapeutic vulnerabilities, including potential dependencies on *CDK5* and *CACNA1D*.^11^ While this study reinforces the clinical relevance of molecular subclassification, it primarily relies on bulk profiling and does not resolve the cellular transcriptional programs that may underpin tumor heterogeneity. Notably, a substantial fraction of pNETs lack canonical driver alterations, leaving key oncogenic mechanisms unresolved.

To address these gaps, we implemented an integrative framework combining bulk and single-nuclei transcriptomic profiling to resolve pNET heterogeneity at high resolution. This approach identified five molecular subtypes with distinct clinical outcomes and uncovered recurrent *BEND2* fusions as central drivers of transcriptional plasticity and tumor aggressiveness. By linking lineage identity, structural alterations, and clinical behavior, this study establishes a molecular taxonomy of pNETs and identifies *BEND2* fusions as defining features of a clinically aggressive subtype, providing a foundation for biomarker-driven stratification and therapeutic targeting.

## Results

### Overview of study cohorts and design

**Figure 1:**
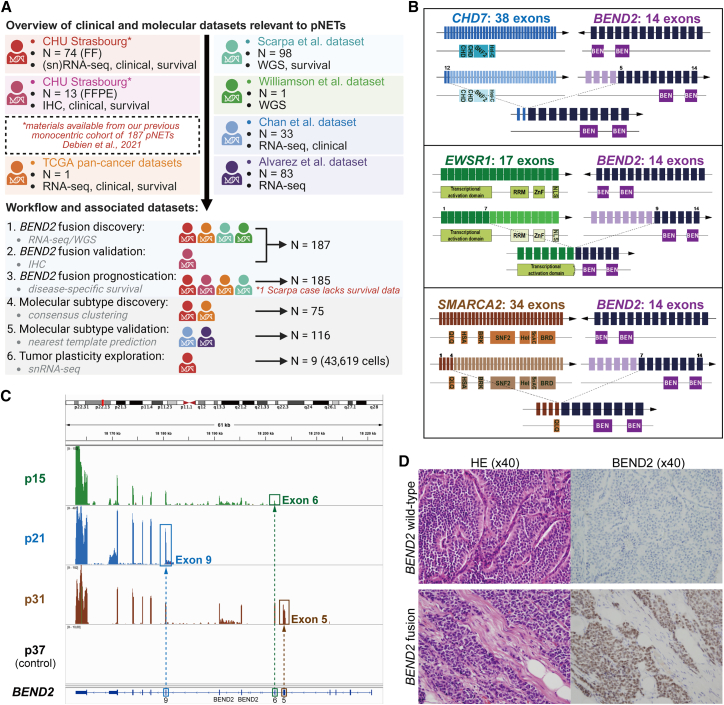
*BEND2* fusion transcripts and expression (A) Flow chart of patient sample collection and cohort description. (B) Illustration of the three fusion transcripts identified by two different pipelines. The complete gene and respective breakpoints are indicated. *EWSR1* is colored in green, *CHD7* in blue, *SMARCA2* in brown, and *BEND2* in purple. The lighter shade of each color corresponds to the lost exons. The predicted fusion transcript is shown, and the protein domains are indicated by the tracks below the transcript. (C) RNA sequencing reads of samples with identified *BEND2* fusion (p21: *CHD7-BEND2*; p31: *EWSR1-BEND2*), *BEND2* expression alone (p15), and a random control case (p37) aligned to the *BEND2* gene. The first conserved exon of *BEND2* is identified by a box matching the color of each fusion partner. (D) Representative pictures of BEND2 immunostaining in one control sample and one sample with *BEND2* fusion.

From our recently described monocentric cohort at Strasbourg University Hospital (CHU Strasbourg),^12^ we performed paired-end RNA sequencing (RNA-seq) on 74 primary pNETs with available fresh-frozen (FF) material. An additional 13 formalin-fixed, paraffin-embedded (FFPE) primary pNET samples from the same cohort, with available tissue and consent, were used for validation. In addition to these two in-house datasets, we incorporated five published datasets of pNETs, resulting in a total of seven datasets encompassing genomic, transcriptomic, immunohistochemical, and single-nucleus RNA-seq (snRNA-seq) data (Figure 1A; Table S1). The study design comprised six sequential components: (1) discovery of *BEND2* fusions using RNA-seq and whole-genome sequencing (WGS) in 187 tumors pooled from five datasets; (2) validation of *BEND2* alterations by immunohistochemistry (IHC); (3) prognostic evaluation of *BEND2* fusions in 185 tumors with available disease-specific survival (DSS) data; (4) identification of transcriptomic subtypes on 75 RNA-seq-profiled tumors; (5) external validation of subtypes in two independent RNA-seq datasets; and (6) exploration of tumor cell plasticity by snRNA-seq.

### Discovery of recurrent *BEND2* fusion transcripts

In the 74 FF pNETs from CHU Strasbourg (Table S2), paired-end RNA-seq identified 21 unique gene fusions supported by > 2 junction reads, including two recurrent *BEND2* fusions (Table S3). One tumor (p21) harbored an in-frame *EWSR1*–*BEND2* fusion (exons 1–7 of *EWSR1* to exons 9–14 of *BEND2*) and another (p31) carried an in-frame *CHD7*–*BEND2* fusion (exons 1–2 of *CHD7* to exons 5–14 of *BEND2*) (Figure 1B). *BEND2* encodes a protein of unknown function containing two C-terminal BEN domains, typically implicated in transcription and chromatin regulation. Both fusions retained these domains, suggesting functional relevance. These cases showed markedly elevated *BEND2* expression compared with the remainder of the cohort, in which expression was undetectable (Figure S1A). A third case (p15) displayed selective expression of the 3′ *BEND2* exons (11–14) retaining BEN domains, without detectable 5′ expression, suggesting the presence of a fusion transcript, although it was not identified by any of our pipelines. Integrative Genomics Viewer analysis confirmed overexpression of the retained exons in fusion-positive cases, with loss of 5′ exons (Figure 1C). IHC demonstrated *BEND2* protein expression in these tumors, with no staining in other pNETs (Figure 1D). To orthogonally validate these findings, we performed RT-PCR followed by qPCR using primers designed from the RNA-seq breakpoints (Figure S1B). Fusion transcripts were confirmed in p21 and p31, whereas p15 and a control tumor (p27) were negative. The absence of amplification in p15, despite high *BEND2* expression, suggests the involvement of an alternative and uncharacterized 5′ fusion partner not targeted by the available primer sets. As expected, paired adjacent normal tissues were consistently negative, confirming tumor-specific expression of the detected fusion transcripts (Figure S1C).

To assess the occurrence of *BEND2* fusions beyond our cohort, we analyzed 10,967 tumors across 32 histological cancer subtypes from The Cancer Genome Atlas (TCGA) and identified only one positive case, annotated as pancreatic ductal adenocarcinoma but pathologically consistent with a grade 2 well-differentiated pNET (Figure S1D). This tumor harbored a *SMARCA2*–*BEND2* fusion, again retaining both BEN domains (Figure 1B; Table S4) and showing the highest *BEND2* expression among all TCGA cases (Figure S1E). Notably, the only ACTH-secreting pNET in our cohort carried an *EWSR1*–*BEND2* fusion (p21). In a validation set of 13 FFPE pNETs from CHU Strasbourg, IHC identified one additional *BEND2*-positive case (Table S5). Overall, *BEND2* alterations were present in 4.6% (4/87) of pNETs analyzed in our in-house datasets.

### Association of *BEND2* fusion with poor clinical outcomes

**Figure 2:**
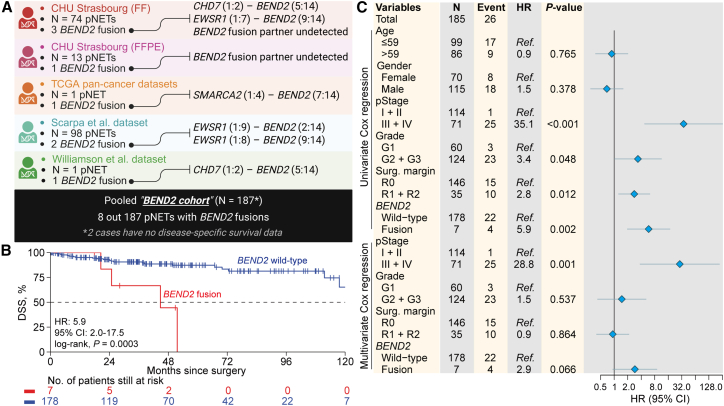
Association of *BEND2* fusions with poor clinical outcomes (A) Diagram of sample composition for the pooled *BEND2* cohort of pNET. (B) Kaplan-Meier curve of disease-specific survival rate regarding *BEND2* rearrangement status. (C) Forest plot showing the hazard ratio (95% CI) in the univariate Cox regression and multivariate regression after adjusting for major clinicopathological features and the corresponding *p* values. The total number for the cohort, the number of cases per variable category, and the number of events (disease-specific deaths) for each level were also indicated.

To assess the clinical relevance of *BEND2* alterations, we analyzed a pooled *BEND2* cohort of 187 pNETs from five datasets, comprising our in-house cases (*n* = 87; four *BEND2* fusions), one fusion-positive case from TCGA, two from the International Cancer Genome Consortium (Scarpa et al.,^13^ *n* = 98), and one from a published case report (Williamson et al.^6^) (Figure 2A). In total, eight tumors harbored *BEND2* fusions. These tumors were strongly associated with advanced disease: six were stage IV and one stage III (7/8 [87.5%] vs. 65/179 [36.3%]; *p* = 0.006), with marked enrichment in metastatic presentations (6/8 [75%] vs. 49/179 [27.4%]; *p* = 0.009) (Table S6). Among patients with metastatic disease (*n* = 55), *BEND2* fusions occurred in 11% of cases, compared with 4.6% in the overall unselected cohort. *BEND2*-altered tumors were associated with significantly shorter DSS (median: 44.8 months vs. not reached; *p* = 0.0003; Figure 2B). In multivariable analysis, *BEND2* alterations showed a trend toward independent prognostic significance after adjustment for major clinical variables (hazard ratio [HR] = 2.9, 95% confidence interval [CI]: 0.9–8.9; *p* = 0.066) (Figure 2C).

### Association between transcriptome subtypes and *BEND2* alterations

**Figure 3:**
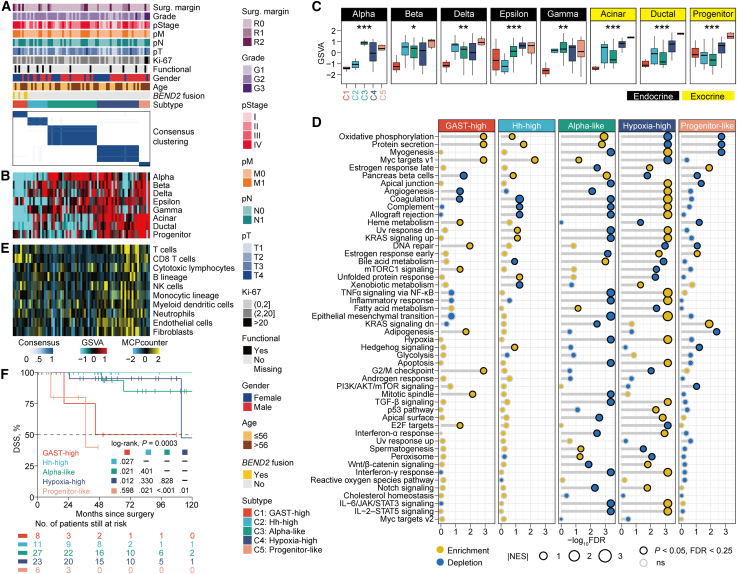
Identification of transcriptomic subtypes (A) Consensus heatmap showing inter-cluster similarity of five transcriptomic subtypes. (B) Heatmap showing enrichment level of major pancreatic cell types quantified by GSVA. (C) Distribution of enrichment level of major pancreatic cell types among five subtypes. (D) Gene set enrichment analysis identified subtype-specific Hallmark features. Each dot describes the enrichment (normalized enrichment score [NES] > 0, *p* < 0.05, FDR <0.25) or depletion (NES <0, *p* < 0.05, FDR <0.25) of each Hallmark gene set. (E) Heatmap showing the relative abundance of 10 immune/stromal cell populations across five subtypes. (F) Kaplan-Meier curves showing different disease-specific survival (DSS) rate among five subtypes.

To dissect the heterogeneity of pNETs, we performed unsupervised consensus clustering on RNA-seq profiles using the top 25% highly variable genes of 75 primary pNETs, comprising 74 FF cases from CHU Strasbourg and one *BEND2*-fusion case from TCGA. Stability assessment supported several plausible solutions (*k* = 3–5) (Figure S2A). Inspection of the consensus matrices and their correspondence across resolutions showed that the *k* = 5 solution provided the most distinct and well-defined consensus block structure (Figures S2B and S2C). We therefore adopted the five-cluster solution for downstream analyses, within which one subtype consisted entirely of *BEND2*-fusion tumors (*n* = 4; 50.3%) (Figure 3A). Principal-component analysis (PCA) using gene expression confirmed clear separation among the five subtypes, while also revealing marked intra-subtype heterogeneity, particularly between subtypes C1–C3 and C4–C5 (Figure S3).

### Transcriptomic similarity to various pancreatic cell types

As mature pancreases consist of a variety of cell types with interdependent functions, we evaluated the transcriptomic similarity of our samples to major pancreatic cell types (Figures 3B and 3C). We found that C2 was predominately characterized by a mixture of beta, delta, and gamma cells, while C3 showed transcriptomic similarity specifically with alpha cells. In addition, C4 and C5 displayed transcriptomic similarity with exocrine cells, including acinar, ductal, and progenitor cells.

To delineate gene markers associated with each subtype, we performed differential expression analysis across clusters (Table S7). In the C1 subtype, gastrin (GAST) was the most significantly overexpressed gene (fold change [FC] = 147, false discovery rate [FDR] <0.0001) (Figure S4A). C2 exhibited elevated expression of several genes associated with the Hedgehog (Hh) signaling pathway, including *KCNH8*, *ADGRG1*, *SLIT1*, *NKX6-1*, *SCG2*, *ETS2*, and *CDK5R1* (all, FC > 2, *p* < 0.05, FDR <0.15) (Figure S4B). Although these are not canonical Hedgehog pathway components, their coordinated upregulation suggests a transcriptional program related to Hedgehog signaling.

Using the top cell-type markers mostly distinguished from each other (mean of normalized count >3, FC > 4, FDR <0.05),^14^ we found that C3 exhibited high expression of eight alpha cell markers, including *RGS4*, *TTR*, *ALDH1A1*, *CRYBA2*, *PCSK2*, *GC*, *TM4SF4*, and *CHGB* (all, FC > 4, FDR <0.05) (Figure S4C). C4 showed overexpression of hypoxia-associated genes (e.g., *ADM*, *PFKFB3*, *LDHA*, *BHLHE40*, *SLC2A3*, *IGFBP3*, and *CP*; all, FC > 2, FDR <0.05) and angiogenesis-relevant genes, including *CA9* (FC = 4.5, FDR = 0.004) and *CALCA* (FC = 4.1, FDR = 0.023) (Figure S4D). C5 exhibited exclusive expression of *GP2* (FC = 295.3, FDR <0.001), a progenitor pancreatic marker, *PTF1A* (FC = 16.8, FDR <0.0001), a transcription factor involved in pancreatic progenitor determination, as well as *GATA4* (FC = 4.6, FDR = 0.04), *HNF1B* (FC = 3.9, FDR = 0.01), and *NR5A2* gene (FC = 6.2, FDR <0.001) (Figure S4E). Notably, C1 exhibited a markedly unbalanced profile of differentially expressed genes, with 2,885 upregulated and only 250 downregulated genes (2,885 vs. 250; FC > 2 or <0.5, FDR <0.05), suggesting widespread transcriptomic dysregulation in the *BEND2*-altered subtype (Figure S4A).

### Biological relevance of transcriptomic subtypes

To gain a deeper understanding of the biological relevance behind each subtype, we further performed gene set enrichment analysis (GSEA) using the Hallmark gene sets (Figure 3D). Our analysis revealed that C1 subtype activated cell-cycle-related pathways, including G2/M checkpoint, E2F targets, mTORC1 signaling, and mitotic spindle, while the C2 subtype showed exclusive activation of the Hedgehog signaling pathway. We also found dysregulation of immune/stromal-related pathways (e.g., inflammatory response, epithelial mesenchymal transition [EMT], TGF-β signaling, interferon-α and interferon-γ signaling pathways) in C3 and C4 subtypes. Additionally, we observed exclusive activation of hypoxia and angiogenesis pathways in the C4 subtype.

Based on these findings, we designated five subtypes from bulk RNA-seq, including a GAST-high subtype characterized by significantly higher expression of *GAST* (C1), an Hh-high subtype enriched for a Hedgehog-related transcriptional program (C2), an Alpha-like subtype with transcriptomic similarity with pancreatic endocrine alpha cells (C3), a Hypoxia-high subtype with exclusive activation of hypoxia and angiogenesis pathways (C4), and a Progenitor-like subtype with transcriptomic similarity with pancreatic progenitor cells including overexpression of the pancreatic progenitor marker *GP2* (C5).

### Tumor microenvironment landscape

Most sporadic pNETs present as well-demarcated solitary masses and are highly vascularized with small vessels and little fibrotic stroma.^15^^,^^16^ Compared to pancreatic ductal adenocarcinomas, pNETs are often characterized by low levels of tumor-infiltrating lymphocytes (TILs), but a specific subset has been previously observed to exhibit higher TIL abundance,^17^ suggesting that the TME had an essential role in pNETs. In this context, we profiled the TME landscape by quantifying the infiltration levels of 10 microenvironment cell types (Figure 3E). Consistent with the GSEA results, Hypoxia-high was highly infiltrated by several immune cells, including T cells, natural killer (NK) cells, monocytic lineage and myeloid dendritic cells (all, *p* < 0.05), and two stromal cells (i.e., endothelial cells and fibroblasts, both *p* < 0.0001) (Figure S5A). The enrichment of endothelial cells in this subtype was also to reconcile with the activation of hypoxia and angiogenesis pathways. Notably, the GAST-high subtype showed a unique enrichment of CD8^+^ T cells (Figure S5B), indicative of a potentially inflamed TME phenotype, whereas other subtypes generally exhibited immune-depleted profiles.

### Aggressiveness of GAST-high and Progenitor-like subtypes

The current molecular classification was prognostic concerning disease-specific survival (DSS; *p* = 0.0003; Figure 3F); median survival was 44.8 months for the GAST-high subtype, 111.6 months for Hypoxia-high subtype, and 37.4 months for the Progenitor-like subtype, whereas median survival was not reached for both Hh-high and Alpha-like subtypes. Specifically, the GAST-high subtype showed significantly poor clinical outcome compared to Hh-high (*p* = 0.027), Alpha-like (*p* = 0.021), and Hypoxia-high (*p* = 0.012) subtypes. Of note, the Progenitor-like subtype also presented with poor prognosis compared to Hh-high (*p* = 0.021), Alpha-like (*p* < 0.001), and Hypoxia-high (*p* = 0.01) subtypes. The inferior prognosis of Progenitor-like may also be due in part to the enrichment of G3 tumors (3/6 [50%] vs. 1/68 [1.5%], *p* = 0.002) and >20% Ki67 (2/6 [33.3%] vs. 0, *p* = 0.006) compared to other subtypes (Table S8). Additionally, we observed a marginally different distribution of gender among five subtypes (*p* = 0.06); specifically, the GAST-high subtype was significantly enriched in female patients as compared to other subtypes (7/8 [87.5%] vs. 24/67 [35.8%], *p* = 0.007) (Table S8). Median DSS for GAST-high and Progenitor-like pNET was 44.8 months while not reached for other pNETs (*p* < 0.001; Figure S6A). Of note, we found that those aggressive (i.e., GAST-high and Progenitor-like) subtypes remained an independent prognostic factor after adjusting other major clinical prognostic features (HR = 15.8, 95% CI: 2.1–118.5, *p* = 0.007) (Figure S6B).

### Immunohistochemical assessment of GAST-high subtype markers

To support the transcriptomic classification, we performed IHC for GAST and CDX2, two markers markedly upregulated at the RNA level in the GAST-high subtype. Both GAST and CDX2 showed strong concordance between RNA and protein expression (*R* = 0.48 and 0.45, respectively; both, *p* < 0.0001; Figure S7A). Importantly, their protein expression was significantly enriched and largely restricted to GAST-high tumors (Figure S7B). These results reinforce the transcriptomic classification and highlight GAST and CDX2 as practical IHC surrogates for identifying the GAST-high subtype in clinical settings.

### Independent validation of bulk transcriptomic taxonomy

To investigate the reproducibility of our subtype’s classification in independent cohorts of pNET, we generated a signature containing the top 200 uniquely and significantly up-regulated mRNAs for each of our five subtypes (FC > 2, FDR <0.05; Figure S8A; Table S9). We performed nearest template prediction (NTP) using the 1,000-gene bulk signature on our cohort and demonstrated high accuracy and reliability of prediction (69 out of 75 [accuracy: 92%], kappa = 0.893, *p* < 0.001; Figure S8B).

We next applied the bulk signature to an external cohort of 33 pNETs (Chan et al.^7^), which also included profiling of *ATRX/DAXX/MEN1* (A-D-M) mutational status. All predicted Alpha-like cases harbored A-D-M mutations (11 [100%] vs. 8/22 [36.4%], *p* = 0.0005; Figure S9A), consistent with prior reports linking alpha-cell-like signatures to A-D-M loss.^7^ For the Progenitor-like subtype, we observed overexpression of pancreas-progenitor-associated genes, including *HNF1B* (FC = 3.1, *p* = 0.001, FDR = 0.19) and *NR5A2* (FC = 4.7, FDR = 0.003). Strikingly, the single case predicted as GAST-high (WT_mk16) was the only tumor expressing *BEND2* (log_2_TPM = 4.1 vs. 0 in others, *p* < 0.0001) and harbored a *NCOA2-BEND2* fusion. This case also exhibited the highest *GAST* expression across the cohort (log_2_TPM = 16 vs. 3.1, *p* = 0.05). As anticipated, the Hypoxia-high subtype demonstrated pronounced TME infiltration, with significant enrichment of endothelial and fibroblast cells (Figure S9A).

We further validated our taxonomy in a larger cohort of 83 primary pNETs (Alvarez et al.^18^) (Figure S9B). Five subtypes were again reproduced, and the Hypoxia-high subtype consistently displayed enriched stromal infiltration. Within this cohort, the only *BEND2*-expressing tumor (AC153, log_2_TPM = 6) harbored an *SSBP3-BEND2* fusion and was classified as GAST-high. *GAST* was exclusively overexpressed in this subtype (FC = 16.7, *p* = 0.0002, FDR = 0.008). In addition, genes defining the Progenitor-like subtype—*GP2* (FC = 262.4, *p* < 0.0001, FDR <0.0001), *PTF1A* (FC = 11.2, *p* = 0.0001, FDR = 0.01), and *NR5A2* (FC = 5.7, FDR = 0.006)—were uniquely overexpressed compared with other subtypes.

Across all datasets with available genomic profiling, we examined the relationship between *BEND2* fusions and canonical pNET drivers (*ATRX*, *DAXX*, and *MEN1*). Strikingly, *BEND2* fusions were mutually exclusive with A-D-M mutations (Figure S9C), indicating that *BEND2* defines an alternative oncogenic mechanism rather than co-occurring with established drivers.

### Inter-tumor heterogeneity at single-nuclei level

**Figure 4:**
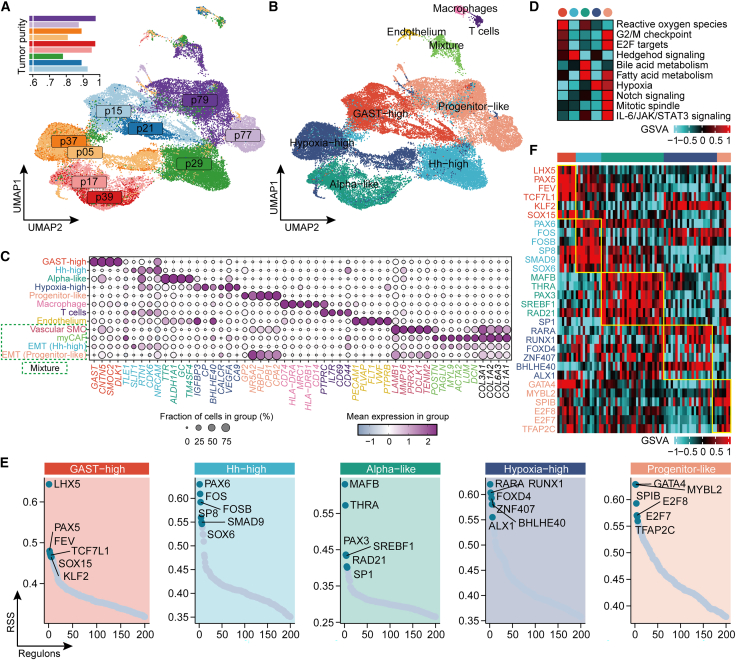
Inter-tumor heterogeneity at single-nuclei level (A) UMAP representation of 43,619 nuclei isolated from nine pNET samples with tumor purity annotated at the top left corner. (B) UMAP showing a separation between tumor cells and non-tumor cells. Tumor cells were grouped according to the five-subtype bulk classification, referred as pseudo-bulk sn-clusters. (C) Dot plot of canonical marker genes across all identified cell populations. Dot size indicates the proportion of cells expressing a gene, and color intensity reflects mean expression levels. (D) Heatmap of the Hallmark signaling pathways specific for each of the five pseudo-bulk sn-clusters based on GSVA enrichment scores. (E) Regulon specificity plot showing the top six regulons identified for each of the five pseudo-bulk sn-clusters. The *x* axis of the plot represents the genes within the regulon, while the *y* axis represents the specificity score. (F) Validation of bulk subtype-specific regulon on the bulk pNET cohort using GSVA based on a set of target genes within a regulon.

While bulk data analysis provides a useful snapshot of gene expression across a population of cells, it may mask important differences between individual cells. In order to validate our bulk data findings and gain a more comprehensive understanding of the cellular heterogeneity within our cohort, we performed snRNA-seq on 11 representative samples covering the entire spectrum of the five bulk subtypes. Of these, nine samples passed the quality control, resulting in sequencing results for 43,619 cells with a median number of 5,001 cells per sample and a median gene count of 1,242 (Table S10). After removing batch effects, the majority of cells were identified as tumor cells, consistent with the high tumor purity of the samples, which ranged from 0.78 to 0.98 (median: 0.89) estimated by bulk RNA-seq data (Figures 4A and 4B). Interestingly, T cells, macrophages, and endothelial cells formed separate clusters compared to tumor cells, and the tumors were grouped according to the five subtypes bulk classification, referred as pseudo-bulk single nucleus (sn)-clusters, exhibiting limited heterogeneity within individual samples (Figure 4B). All cell populations displayed high data quality, with adequate gene detection and unique molecular identifier (UMI) counts (Figure S10).

We identified consistent gene markers for each sn-cluster, reinforcing the subtype definitions derived from bulk transcriptomic profiling (Figure 4C). The GAST-high sn-cluster was defined by high expression of *GAST*. The Hh-high sn-cluster showed elevated expression of Hedgehog-pathway-associated genes, including *TLE1* and *SLIT1*, which were also enriched in the corresponding bulk subtype, together with additional pathway-related genes overexpressed at the single-nuclei level (*RTN1*, *CDK6*, and *NRCAM*). The Alpha-like sn-cluster expressed key alpha cell markers such as *TTR*, *ALDH1A1*, *GC*, and *TM4SF4*, while the Hypoxia-high sn-cluster was marked by upregulation of hypoxia-associated genes (*BHLHE40*, *IGFBP3*, and *CP*) and angiogenesis-related genes (*CA9*, *CALCR*, and *VEGFA*), consistent with the pathway enrichment (Figure S11). The Progenitor-like sn-cluster exhibited co-expression of pancreatic progenitor markers such as *GP2* and *NR5A2*, along with *RBPJL*, *CPA1*, and *CPA2*, consistent with a late acinar-like differentiation state.

When we projected these snRNA-seq markers onto our bulk RNA-seq dataset, they recapitulated distinct subtype-specific expression patterns (Figure S12A). Moreover, classification of single-nucleus transcriptomes using bulk RNA-derived signatures resulted in strong concordance with pseudo-bulk sn-clusters (Kappa coefficient: 0.733, Fisher’s exact test *p* < 0.001; Figures S12B and S12C). An exception was the Hypoxia-high sn-cluster, which aligned with the Alpha-like bulk signature—likely due to high endothelial cell content in bulk samples, diluting the tumor-intrinsic hypoxia signal (Figure S12D).

To further characterize pathway activity across snRNA-seq subtypes, we conducted hallmark GSEA. Both GAST-high and Progenitor-like clusters exhibited enrichment for proliferation-associated programs, including E2F targets and G2/M checkpoint pathways. The Progenitor-like cluster additionally showed enrichment in Notch signaling. The Hypoxia-high cluster demonstrated upregulation of the hypoxia pathway, and the Alpha-like cluster showed activation of fatty acid metabolism. The Hh-high cluster exhibited a transcriptional program co-expressed with Hedgehog-pathway-related genes, suggesting potential engagement of a non-canonical or context-dependent Hedgehog-like state at the tumor-cell level (Figure 4D).

To assess the transcription factor (TF) network associated with each pseudo-bulk sn-clusters, we performed SCENIC analysis and identified distinct regulatory networks (regulons) across different subtypes (Figures 4E and 4F). GAST-high was associated with activation of the FEV regulon, which has been shown to regulate endocrine progenitor cells.^19^ In addition, we observed the activation of LHX5 regulon, a member of LIM homeobox family of TF, whose spatiotemporal expression patterns define distinct anatomical compartments during central nervous system development.^20^ Hh-high was associated with *PAX6*, which maintains beta and gamma cell identity. Alpha-like was associated with *MAFB*, a specific alpha cell marker. Hypoxia-high was enriched for *RARA*, *RUNX1*, and *BHLHE40*, the last of which is a transcriptional repressor whose activation has been reported to cause pancreatic β-cell dysfunction and subsequently lead to hypoxia.^21^ Progenitor-like was associated with the *GATA4* regulon, whose expression in the pancreatic endoderm becomes restricted to the exocrine compartment. This is in contrast to *GATA6*, which remains restricted to the endocrine compartment.^22^ Furthermore, the Progenitor-like subtype was linked to regulons associated with proliferation, such as *MYBL2* and *E2F7*.

### Tumor and microenvironmental diversity include EMT-like and stromal phenotypes

In addition to the five major transcriptomic subtypes, we identified a transcriptionally heterogeneous cluster composed of diverse minor cell populations (Figure 4B). Sub-clustering of this population revealed four transcriptionally distinct sub-clusters, including myofibroblastic-cancer-associated fibroblasts (myCAFs), vascular smooth muscle cells, and two rare subsets of tumor cells exhibiting features of EMT (Figures S13A and S13B). These EMT-like cells expressed robust levels of collagen genes (*COL1A1* and *COL1A2*) and retained subtype-specific markers (Figure 4C), suggesting the presence of an EMT-like neuroendocrine state rather than stromal contamination.

To evaluate whether tumor cell composition at the cellular level reflects the molecular subtypes identified by bulk profiling, we applied cell-type-specific expression profiles derived from our snRNA-seq data to deconvolute bulk RNA-seq samples and estimate the relative proportion of each cell population (Figures S14A and S14B; Table S11). Although based on a limited number of snRNA-seq samples, the inferred cell-type fractions showed patterns broadly consistent with bulk-defined subtypes. For example, patients classified as GAST-high or Progenitor-like subtypes exhibited higher estimated proportions of GAST-high (48% vs. 1%) and Progenitor-like (88.8% vs. 4%) tumor cells, respectively, compared with other subtypes. Similarly, Alpha-like tumors were enriched for Alpha-like cells (59.8% vs. 20.2%), Hypoxia-high tumors for Hypoxia-high cells (29% vs. 10.3%), and Hh-high tumors for Hh-high cells (6.3% vs. 0%) (Figure S14C). Notably, a higher inferred proportion of GAST-high tumor cells (HR = 21.5, 95% CI: 2.4–190.5, *p* = 0.006) or Progenitor-like tumor cells (HR = 13.3, 95% CI: 1.7–115.8, *p* = 0.019) was associated with worse DSS (Table S12), supporting the potential clinical relevance of subtype-specific cellular composition.

### Identification of therapeutic targets through cell-cell communication

**Figure 5:**
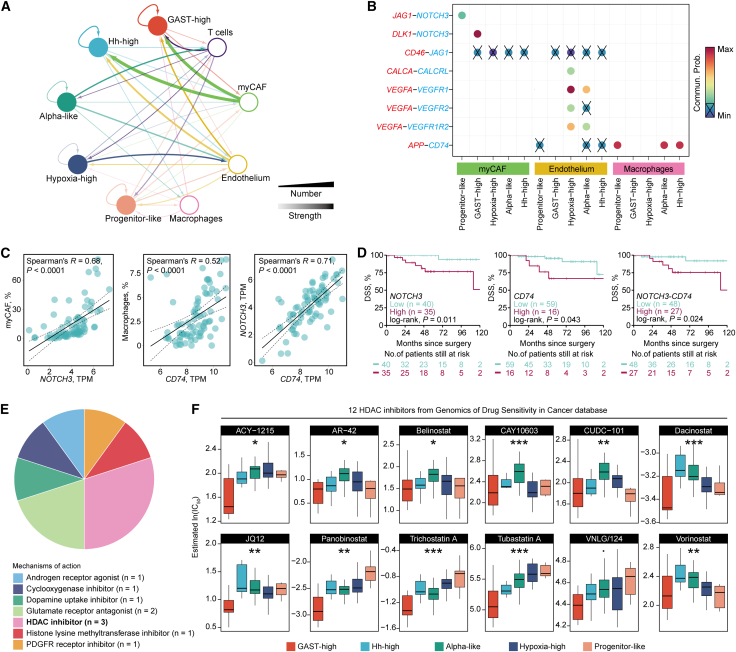
Cell-cell communication network and *in silico* drug sensitivity prediction in pNET subtypes (A) Cell-cell communication network visualized in Cytoscape, depicting the number and strength of interactions between tumor and microenvironmental cell populations. Nodes represent cell types, and edges represent intercellular interactions, with edge width and color reflecting interaction strength and frequency. (B) Bubble plot of selected ligand-receptor pairs between tumor subtypes and non-tumor microenvironmental cell populations. (C) Correlation plots showing associations between gene expression and deconvoluted cell-type proportions in bulk pNET samples. (D) Kaplan-Meier survival curves showing different DSS rates between pNET patients with high and low expression of genes of interest, including *NOTCH3* (left), *CD74* (middle), and the geometric mean value of these two genes (right). Patients were categorized into different groups according to the optimal thresholding. (E) Mechanisms of action of the 10 compounds predicted by Connectivity Map (CMap) analysis to preferentially target the *BEND2* fusion. (F) Predicted sensitivities to 12 HDAC inhibitors across pNET subtypes based on GDSC database analyses. Statistical comparisons were performed using the Kruskal-Wallis test. *p* < 0.1; ∗*p* < 0.05; ∗∗*p* < 0.01; ∗∗∗*p* < 0.001.

To identify subtype-specific microenvironmental dependencies and therapeutic vulnerabilities, we analyzed intercellular communication patterns within each pseudo-bulk sn-cluster. Among the stromal and immune cell types, myCAFs exhibited the most extensive interactions with tumor cells across all subtypes, surpassing those of endothelial and immune cells (Figure 5A). Notably, cancer cells in the Hypoxia-high and Alpha-like clusters displayed strong crosstalk with endothelial cells, while Hh-high tumor cells showed predominant interactions with macrophages. Further analysis of ligand-receptor interactions revealed that tumor cells from aggressive subtypes engaged in strong signaling with myCAFs through the *JAG1*–*NOTCH3* and *DLK1*–*NOTCH3* axes, and with macrophages via *APP*–*CD74* (Figure 5B). Deconvolution of bulk RNA-seq data showed that the inferred abundance of myCAFs was strongly correlated with *NOTCH3* expression (*R* = 0.68), and macrophage proportion correlated with *CD74* expression (*R* = 0.52) (Figure 5C). Expression levels of *NOTCH3* and *CD74* were also highly correlated (*R* = 0.71), and high expression of either gene—or a composite *NOTCH3*–*CD74* signature—was significantly associated with poor DSS (all, *p* < 0.05; Figure 5D). These findings suggest that targeting *NOTCH3* and *CD74* may be a potential therapeutic strategy for aggressive pNET subtypes.

### *In silico* prediction of drug sensitivities in BEND2-fusion pNETs

Given the enrichment of *BEND2* fusions within the GAST-high subtype and the chromatin-associated role of *BEND2*, we next investigated whether this subtype might harbor selective vulnerabilities to epigenetic therapies. Using the Connectivity Map (CMap), we identified 10 compounds predicted to preferentially target *BEND2*-fusion tumors, encompassing seven distinct mechanisms of action (Figure 5E). Notably, histone deacetylase (HDAC) inhibitors appeared in three independent perturbations, suggesting that their occurrence was unlikely to be random and supporting epigenetic modulation as a plausible therapeutic avenue. To further substantiate this finding, we used the Genomics of Drug Sensitivity in Cancer (GDSC) database to predict sample-level responses across 12 HDAC inhibitors. Predicted IC_50_ values revealed that most HDAC inhibitors displayed greater sensitivity in the GAST-high subtype compared to other clusters (Figure 5F). Together, these results suggest that *BEND2*-fusion tumors may be particularly susceptible to HDAC inhibition, providing a rationale for therapeutic stratification in this aggressive subtype.

### *BEND2* fusion induces transcriptional reprogramming and mesenchymal morphology in human pNET cells

**Figure 6:**
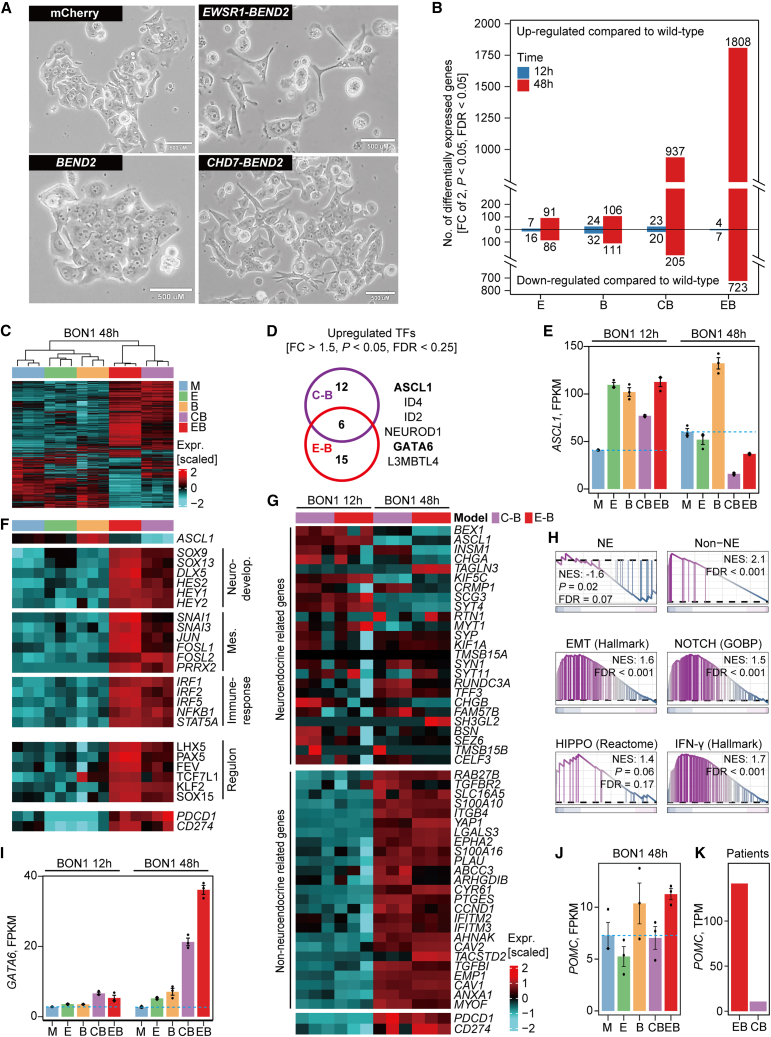
*BEND2* fusions induce transcriptional reprogramming and morphological plasticity in pNET tumor cells (A) Phase-contrast microscopy images of BON1 cells at 48 h post-induction showing morphological changes following overexpression of *BEND2*-only, *CHD7*-*BEND2*, *EWSR1*-*BEND2*, or mCherry control. (B) Bar plot showing the number of differentially expressed genes at 12 and 48 h across all BON1 cell line models, including *EWSR1* (E), *BEND2* (B), *CHD7*-*BEND2* (CB), and *EWSR1*-*BEND2* (EB), compared to the mCherry (M) control. (C) Heatmap illustrating transcriptomic clustering of BON1 cell lines at 48 h (D) Venn diagram showing overlapping significantly upregulated transcription factors (TFs) in *CHD7*-*BEND2* and *EWSR1*-*BEND2* models compared to controls at 12 h (E) Bar plot of *ASCL1* expression across BON1 cell line models at 12 and 48 h. Data are represented as mean ± SEM. (F) Heatmap showing transcriptional activation of neurodevelopmental, mesenchymal, and immune-related TFs in fusion-expressing BON1 cells at 48 h, accompanied by *ASCL1* downregulation, activation of GAST-high subtype-specific regulons, and upregulation of immune checkpoint genes *PDCD1* and *CD274*. (G) Heatmap illustrating temporal transcriptomic shifts in BON1 fusion models using a 50-gene classifier (25 neuroendocrine and 25 non-neuroendocrine genes) derived from human SCLC lines. (H) GSEA results showing transcriptional reprogramming at 48 h in *BEND2* fusion lines compared to 12 h (I) Bar plot showing *GATA6* expression uniquely and robustly upregulated in *BEND2* fusion lines at both 12 and 48 h. Data are represented as mean ± SEM. (J) Bar plot showing specific upregulation of *POMC* in BON1 cells expressing *EWSR1*-*BEND2* at 48 h. Data are represented as mean ± SEM. (K) Bar plot showing markedly higher *POMC* expression in the *EWSR1*-*BEND2*-positive tumor compared to the *CHD7*-*BEND2*-positive tumor in the clinical cohort.

To investigate the functional impact of *BEND2* fusions, we established doxycycline-inducible overexpression models in the human pNET cell line BON1, including mCherry, overexpression of *BEND2* alone, *EWSR1* alone, the *EWSR1*-*BEND2* fusion, and the *CHD7*-*BEND2* fusion (Table S13). Cells were harvested at 12 and 48 h post-induction for transcriptomic profiling. Successful expression of transgenes and fusion constructs was confirmed by qPCR and bulk RNA-seq (Figures S15A and S15B), and BEND2 protein overexpression was further validated by western blotting (Figure S15C). Morphologically, fusion-positive cells displayed heterogeneity consistent with pooled, rather than clonal, populations: while some retained compact, epithelial-like clusters, others exhibited elongated and spread morphologies reminiscent of mesenchymal-like states, similar to phenotypes previously reported in MYC-driven, non-neuroendocrine subtypes of small cell lung cancer (Figure 6A).^23^ To assess functional consequences, we performed anchorage-dependent colony formation and CellTrace-based proliferation assays. BON1 cells overexpressing *BEND2* or *CHD7*-*BEND2* formed significantly more colonies and exhibited enhanced proliferation compared to controls (Figure S15D). By contrast, *EWSR1* overexpression modestly reduced colony formation, an effect that was more pronounced in *EWSR1*-*BEND2* fusion cells, which also showed reduced proliferation, consistent with an oncogene-induced senescence (OIS) phenotype (Figure S15E). Supporting this, transcriptomic profiling revealed marked upregulation of *CDKN1A* (p21; FC = 5.4, FDR <0.001), a canonical mediator of OIS, in *EWSR1*-*BEND2*-expressing cells compared to others (Figure S15F).

Transcriptomic analysis at 12 h showed minimal differential gene expression across all models compared to mCherry control cells (Figure 6B). In contrast, at 48 h, the *EWSR1*-*BEND2* and *CHD7*-*BEND2* fusion cells exhibited substantial transcriptomic reprogramming, with 2,531 (1808 up, 723 down) and 1,142 (937 up, 205 down) differentially expressed genes, respectively (Figure 6B). Notably, upregulated genes outnumbered downregulated genes (2.5-fold in *EWSR1*-*BEND2* and 4.6-fold in *CHD7*-*BEND2*), mirroring the transcriptional activation observed in the GAST-high subtype, where upregulated genes vastly exceed downregulated ones (Figure S3A). Unsupervised clustering confirmed that mCherry *BEND2*-only and *EWSR1*-only cells clustered together, while both fusion-expressing lines formed a distinct cluster (Figure 6C).

Despite limited changes at 12 h, RNA levels of six TFs were significantly upregulated in both fusion models compared to mCherry control cells (Figure 6D). Among them, *ASCL1*, a key regulator of neuroendocrine differentiation, was notably elevated at 12 h with an FC of 2.7 in *EWSR1*-*BEND2* and 1.9 in *CHD7*-*BEND2* (both FDR <0.05) (Figure 6E). However, by 48 h, *ASCL1* expression was significantly reduced in both fusion models, with an FC of 0.6 in *EWSR1*-*BEND2* and 0.28 in *CHD7*-*BEND2* (both FDR <0.05) (Figure 6E). Concurrently, 48-h profiles of the fusion models showed broad upregulation of TFs involved in neurodevelopmental (*SOX9*, *SOX13*, *DLX5*, and *HES*/*HEY* family), mesenchymal (*SNAI1*, *SNAI3*, *JUN*, *FOSL1*/*2*, and *PRRX2*), and immune-response (*IRF1*, *IRF2*, *IRF5*, *NFKB1*, and *STAT5A*) programs (Figure 6F). Consistent with these signatures, western blot analyses at 48 h confirmed increased SNAIL and SOX9 expression in *CHD7*-*BEND2* cells (Figure S16). Interestingly, SOX9 was also upregulated in *EWSR1*-alone cells, consistent with RNA-seq data (Figure 6F). These orthogonal findings reinforce fusion-driven induction of mesenchymal and neurodevelopmental programs. Regulon analysis using GAST-high subtype-specific TF networks revealed significant activation of regulons such as LHX5, PAX5, FEV, TCF7L1, KLF2, and SOX15 (Figure 6F), supported by gene-level upregulation of *LHX5*, *KLF2*, *FEV*, and *TCF7L1* (Figure S15G). Fusion-positive cells also showed elevated expression of PD-1 (*PDCD1*) and PD-L1 (*CD274*) (Figure 6F), indicative of an inflamed phenotype consistent with CD8^+^ T cell enrichment seen in GAST-high tumors.

Given parallels to SCLC, where *ASCL1*-driven neuroendocrine (NE) states transition to non-NE subtypes,^24^ we applied a 50-gene classifier (25 NE and 25 non-NE markers) derived from human SCLC lines.^25^ Fusion expression promoted a clear shift from neuroendocrine (e.g., *ASCL1*, *INSM1*, and *CHGA*) to non-neuroendocrine (e.g., *YAP1*, *TGFBR2*, and *CCND1*) transcriptional signatures (Figure 6G), further supported by GSEA enrichment of NOTCH, HIPPO, EMT, and interferon-γ response pathways (Figure 6H). Notably, the upregulation of PD-1 and PD-L1 in parallel with *ASCL1* suppression mirrors the inflamed phenotype observed in the mesenchymal subtype of SCLC (Figure 6G), where checkpoint inhibitors show efficacy despite immunosuppressive microenvironments.^24^

Interestingly, one of the few transcription factors upregulated at 12 h was *GATA6*, a known pioneer factor that facilitates chromatin remodeling.^26^ Although global transcriptional changes were minimal at this early time point, *GATA6* was significantly upregulated in both fusion-positive models (FC > 1.5, *p* < 0.05, FDR <0.25) but showed only modest changes in the *BEND2*-only and *EWSR1*-only controls (FC ≤ 1.2). Notably, *GATA6* expression remained strongly elevated at 48 h in the fusion-expressing lines, with an FC of 10.1 in *EWSR1*-*BEND2* and 6.0 in *CHD7*-*BEND2*, compared to only 2.2 and 1.7 in the *BEND2*-only and *EWSR1*-only models, respectively (Figure 6I). The early induction and sustained overexpression of *GATA6* may contribute to the transient upregulation and subsequent downregulation of *ASCL1*, reflecting a shift from a neuroendocrine-associated program toward a more dedifferentiated or lineage-plastic state. These findings suggest that *GATA6* may serve as a pioneer TF in chromatin priming, enabling broader transcriptional reprogramming and facilitating the transition away from classical neuroendocrine identity.

To validate these findings, we tested the QGP1 pNET cell line overexpressing *BEND2*, *EWSR1*, and *EWSR1*-*BEND2* for 48 h (Figure S17A). Consistent with results in BON1 cells, the *EWSR1*-*BEND2* fusion induced a markedly higher number of differentially expressed genes compared to the *BEND2*-only and *EWSR1*-only controls (Figure S17B). This was accompanied by downregulation of *ASCL1* (Figure S17C), upregulation of *GATA6* (Figure S17D), and increased expression of *SOX9*, *DLX5*, *HEY2*, *SNAI1*, *JUN*, *FOSL1*, *PRRX2*, and *IRF1*, as well as subtype-specific regulons (Figures S17E and S17F). Enrichment of non-NE, EMT, HIPPO, NOTCH, and interferon-γ pathways, as well as GAST-high regulon activation, further supported the robustness of the reprogramming (Figure S17E).

Strikingly, *POMC* was among the top upregulated genes in *EWSR1*-*BEND2*-expressing QGP1 cells (FC = 12.4, FDR <0.001; Figure S17G), and also uniquely elevated in the BON1 *EWSR1*-*BEND2* model (FC = 1.5, FDR = 0.04), but not in *CHD7*-*BEND2* or fusion-negative lines (all, *p* > 0.15, FDR >0.4) (Figure 6J). In our clinical dataset, the tumor harboring *EWSR1*-*BEND2* showed 13-fold higher *POMC* expression than the *CHD7*-*BEND2*-positive case (Figure 6K). This supports a specific role for *EWSR1*-*BEND2* in activating *POMC* transcription and aligns with a recent report identifying *EWSR1-BEND2* fusions in two out of four patients with ACTH-secreting pNETs.^27^

## Discussion

This study delineates a molecular taxonomy of pNETs anchored by recurrent *BEND2*-fusion-driven transcriptional reprogramming and its clinical relevance, expanding the spectrum of *BEND2* alterations in cancer.^28^^,^^29^ By integrating bulk and single-nuclei transcriptomic analyses, we classify pNETs into five molecular subtypes and unravel the mechanistic underpinnings of *BEND2* fusions in driving tumor aggressiveness. These findings advance understanding of pNET heterogeneity and inform precision oncology strategies.

*BEND2* encodes an X-linked chromatin-associated protein containing BEN domains involved in transcriptional regulation and chromatin remodeling.^30^^,^^31^ Native expression of *BEND2* is largely restricted to testicular germ cells, and its role in tumorigenesis remains poorly understood.^32^ Previous reports identified *BEND2* fusions in rare central nervous system and soft tissue tumors.^5^^,^^28^^,^^29^^,^^33^^,^^34^^,^^35^ Here, we demonstrate *BEND2* fusions in ∼5% of pNETs (11% in metastatic disease), uniformly associated with high *BEND2* expression, advanced stage, and poor survival. Although our cohort lacked matched DNA-level data, the genomic basis of *BEND2* fusions is supported by prior studies. Scarpa et al. identified *EWSR1-BEND2* genomic rearrangements via WGS in two pNET cases,^5^ and Williamson et al. reported a similar event with concordant RNA-seq evidence.^6^ These findings reinforce the notion that BEND2 fusion transcripts reflect authentic genomic rearrangements.

Mechanistically, *BEND2* fusions induce a dynamic transcriptional cascade characterized by transient upregulation of neuroendocrine lineage factors such as *ASCL1,* followed by their suppression, concomitant with activation of mesenchymal (*SNAI1*, *JUN*), neurodevelopmental (*SOX9*, *DLX5*), and immune-related (*IRF1*, *STAT5A*) programs. These shifts are associated with mesenchymal morphology, a feature often linked to more aggressive and treatment-resistant phenotypes. Interestingly, *EWSR1* overexpression alone led to SOX9 induction, raising the possibility that the fusion partner may contribute to lineage reprogramming independently of *BEND2*. Critically, the sustained overexpression of *GATA6*, a pioneer TF implicated in chromatin remodeling, appears to prime tumor cells for this plasticity. These dynamics mirror mechanisms observed in SCLC, where MYC-driven Notch activation reprograms tumors from *ASCL1*+ neuroendocrine (SCLC-A) to *NEUROD1*+/*YAP1*+ non-neuroendocrine states through transient *ASCL1* suppression and epigenetic rewiring, enabling therapy resistance.^23^^,^^24^^,^^36^ In both malignancies, transient activation of lineage-defining transcription factors (e.g., *ASCL1*) precedes chromatin remodeling and transcriptional plasticity that drive subtype switching. While SCLC evolution is orchestrated by MYC-Notch axis activation, pNETs employ *BEND2* fusions as master regulators of a hybrid transcriptional state bridging neuroendocrine and non-neuroendocrine identities—a paradigm shift in understanding pNET biology.

These findings nominate lineage- and chromatin-targeted therapies as potential vulnerabilities. For instance, DLL3-engaging agents, which show efficacy in ASCL1-high SCLC-A subtypes, could potentially exploit residual neuroendocrine priming in early-phase *BEND2*-positive tumors.^24^ Similarly, HDAC inhibitors, which modulate chromatin remodeling, may help stabilize differentiation states and prevent transitions to more aggressive phenotypes.^24^ This concept is supported by prior studies demonstrating HDAC activity in pNETs,^37^^,^^38^ and by clinical evidence of disease stabilization in pNET patients treated with panobinostat in a phase II trial,^39^ suggesting that chromatin-modifying agents may offer therapeutic benefit in tumors with high transcriptional plasticity. Although direct targeting of *BEND2* remains elusive, downstream nodes such as *GATA6* or *NOTCH3* offer actionable surrogates.

The five molecular subtypes we defined—GAST-high, Hh-high, Alpha-like, Hypoxia-high, and Progenitor-like—stratify patients into clinically prognostic groups. GAST-high and Progenitor-like subtypes represent categories not previously described using transcriptomic, enhancer, or recent proteomic profiling efforts.^8^^,^^10^^,^^11^ The GAST-high subtype, enriched for *BEND2* alterations, is characterized by marked overexpression of *GAST*, the gene encoding gastrin. While gastrin overexpression in pNETs does not always result in functional syndromes, it has been linked to worse prognosis.^2^ Kim et al. also showed that the 5-year survival rate for patients with gastrin-positive pNETs was 40% compared to 82.9% for patients with gastrin-negative pNETs.^40^

The Hh-high subtype, named for its consistent enrichment of Hedgehog-pathway-associated genes across both bulk and snRNA-seq analyses, appears to reflect a non-canonical or developmentally regulated Hedgehog-like transcriptional program. Notably, canonical Hedgehog components such as SHH, PTCH1/2, and GLI1 were not prominently expressed in this subtype. Nonetheless, the reproducibility of this enrichment across platforms and its distinct clustering pattern indicate underlying biological relevance that merits further investigation.

The Progenitor-like and GAST-high subtypes reflect early and late endocrine progenitor states, respectively. The Progenitor-like subtype, which is enriched for high-grade tumors, exhibited acinar/ductal transcriptional signatures and GATA4 regulon activity. In contrast, the GAST-high subtype showed activation of the FEV regulon, a marker of late endocrine progenitors.^41^ *FEV* expression is induced downstream of *NEUROG3* during pancreas development,^42^ and *FEV*+ cells have been described in the fetal pancreas, in human ESC-derived Eps, and in immature endocrine cells.^43^ Clinically, these subtypes stratify patients into distinct prognostic groups, with GAST-high and Progenitor-like tumors exhibiting inferior survival independent of traditional grading systems. This reinforces the need to integrate molecular subtyping into clinical practice, particularly for identifying high-risk patients who may benefit from intensified surveillance or other therapies.

At single-nucleus resolution, we further uncovered distinct multicellular ecosystems associated with each subtype. GAST-high and Progenitor-like tumors exhibited extensive stromal interactions, particularly with myCAFs via *JAG1/DLK1-NOTCH3* signaling, while Hypoxia-high tumors showed endothelial-cell-driven angiogenesis. The correlation between *NOTCH3/CD74* expression and poor survival nominates these pathways as therapeutic targets. Notably, GAST-high tumors displayed paradoxical CD8^+^ T cell enrichment alongside *PD-1/PD-L1* upregulation, suggesting an inflamed but immunosuppressed microenvironment. This duality mirrors the SCLC inflamed/mesenchymal (SCLC-I) subtype, where immune checkpoint inhibitor efficacy is heightened despite microenvironmental immunosuppression, highlighting shared vulnerabilities across neuroendocrine malignancies.^24^ By contrast, Hypoxia-high tumors may benefit more from antiangiogenic strategies such as sunitinib—a hypothesis warranting prospective validation.

Our findings also contribute to the understanding of hormone-producing pNETs. Previous reports have identified *EWSR1*–*BEND2* fusions in approximately 50% of ACTH-producing pNETs compared with ∼2% in unselected cases,^27^ suggesting a possible enrichment of these fusions in this functional subtype. In our cohort, functional status was available for a subset of *BEND2*-positive cases, among which one ACTH-secreting tumor harbored an *EWSR1*–*BEND2* fusion and showed functional consequence: robust upregulation of *POMC*, the ACTH precursor. These findings suggest a possible link between *BEND2* fusions and ectopic ACTH production, providing a plausible mechanistic basis for paraneoplastic Cushing syndrome and supporting the potential utility of fusion testing in the diagnostic evaluation of ACTH-secreting tumors.^44^^,^^45^

In summary, this study defines a molecular framework for pNETs that transcends histopathologic classification. *BEND2*-fusion-driven transcriptional plasticity and high-risk molecular subtypes illuminate tumor evolution, microenvironmental dependencies, and actionable signaling nodes. These findings support integrating *BEND2* screening and molecular subtyping into clinical practice to refine prognosis and personalize therapeutic strategies in pNETs.

### Limitations of the study

Several limitations should be acknowledged. First, the rarity of *BEND2*-altered tumors constrained our sample size and necessitates validation in larger, prospective cohorts. Second, functional status was available for only a subset of *BEND2*-positive cases, with only one ACTH-secreting tumor identified. While consistent with prior reports of *EWSR1*–*BEND2* enrichment in ACTH-producing pNETs, our dataset alone does not permit a statistically robust assessment of this association. Third, although snRNA-seq enabled high-resolution cellular profiling, the limited number of tumors and uneven patient-level representation of certain populations—such as EMT-like tumor cells, myCAFs, and vascular smooth muscle cells—limit their generalizability at the cohort level. These populations, while transcriptionally coherent and passing quality control, were supported by small cell numbers and, in some cases, dominated by individual tumors; they should therefore be interpreted as patient-skewed, hypothesis-generating states requiring validation in larger cohorts and/or orthogonal approaches, including spatial transcriptomics or multiplexed imaging. Fourth, although BON1 and QGP1 are widely employed human pNET-derived cell lines and provide tractable systems, both diverge from primary tumors in growth kinetics and differentiation states. Accordingly, our functional findings serve as proof of concept for *BEND2*-driven transcriptional reprogramming but may not fully capture the indolent biology of pNETs *in vivo*, and translation of candidate therapeutic targets (e.g., *NOTCH3* and *GATA6*) will require validation in more physiologically relevant models.

## Resource availability

### Lead contact

Further information and request for resources should be directed to and will be fulfilled by the lead contact, Gabriel G. Malouf (maloufg@igbmc.fr).

### Materials availability

This study did not generate new unique reagents.

### Data and code availability

• Clinical patient sample metadata and cell line information are provided in the supplementary materials. Pre-processed RNA-seq data from clinical samples and cell lines, along with raw single-nucleus RNA-seq count matrices and associated cell metadata, have been deposited in a public repository on Mendeley Data at https://data.mendeley.com/datasets/r9m66rjtxy/1 (https://doi.org/10.17632/r9m66rjtxy.1).
• This study did not generate custom code. All analyses were performed using publicly available software packages as listed in the key resources table with parameters detailed in the STAR Methods. Analysis scripts and computational workflows used in this study are available from the lead contact upon request.
• Any additional information required to reanalyze the data reported in this work paper is available from the lead contact upon request.

## Acknowledgments

We would like to express our deepest gratitude to all the patients whose data contributed to this study. We thank BioRender (BioRender.com) for providing the platform to create graphical abstract and flow diagrams. This work was supported in part by grants from the 10.13039/100016913MSDAVENIR research grant, Groupe d’étude des Tumeurs neuroendocrines (GTE).

## Author contributions

Conceptualization, X.L., P. Baltzinger, L.X., S.K.B., and G.G.M.; methodology, X.L., P. Baltzinger, L.X., S.K.B., and F.A.; formal analysis, X.L., L.X., and S.K.B.; investigation, all authors; resources, M.-P.C., P. Baltzinger, P. Bachellier, P.A., and A.F.; data curation, X.L., P. Baltzinger, L.X., S.K.B., W.C., V.D., C.V., and X.S.; writing—original draft, X.L., P. Baltzinger, L.X., S.K.B., and G.G.M.; writing—review & editing, all authors; visualization, X.L., L.X., P. Baltzinger, A.F., S.K.B., and A.F.; supervision, J.-E.K., I.D., X.S., B.G., S.K.B., and G.G.M.; project administration, X.S., B.G., and G.G.M.; funding acquisition, G.G.M.

## Declaration of interests

The authors declare no competing interests.

## STAR★Methods

### Key resources table

**Table:** 

REAGENT or RESOURCE	SOURCE	IDENTIFIER
**Antibodies**		

Rabbit polyclonal anti-BEND2 (CXorf20)	Abcam	Cat# ab204795
Rabbit anti-CDX2	Zytomed Systems	Cat# RBK019-05
Rabbit polyclonal anti-Gastrin	Cell Marque	Cat# 256A14
Anti-Actin (loading control)	Sigma-Aldrich	Cat# A5441

**Bacterial and virus strains**		

Lentiviral particles: pLenti-TetON-FLAG-HA-mCherry	This paper (sequence synthesized by GenScript)	N/A
Lentiviral particles: pLenti-TetON-FLAG-HA-*BEND2*	This paper (sequence synthesized by GenScript)	N/A
Lentiviral particles: pLenti-TetON-FLAG-HA-*EWSR1*	This paper (sequence synthesized by GenScript)	N/A
Lentiviral particles: pLenti-TetON-FLAG-HA-*CHD7-BEND2*	This paper (sequence synthesized by GenScript)	N/A
Lentiviral particles: pLenti-TetON-FLAG-HA-*EWSR1-BEND2*	This paper (sequence synthesized by GenScript)	N/A

**Biological samples**		

Human tumors of CHU Strasbourg	This study	N/A
BON1 (human pNET cell line)	Kind gift of M. Heaphy	CVCL_3985
QGP1 (human pNET cell line)	Kind gift of M. Heaphy	CVCL_3143

**Chemicals, peptides, and recombinant proteins**		

Polyethylenimine (PEI)	Polysciences	Cat# 23966
Lipofectamine™ 2000 Transfection Reagent	Invitrogen	Cat# 11668027
Opti-MEM™ I Reduced Serum Medium	Thermo Fisher Scientific	Cat# 31985062
Protease Inhibitor Cocktail	Sigma-Aldrich	Cat# 40091500
Bradford Reagent (Protein Assay Dye)	Bio-Rad	Cat# 5000006
ECL reagent	Protein Biology	Cat# UC180107
NuPAGE 4–12% Bis-Tris Protein Gel	Invitrogen	Cat# 20070610
CellTrace™ Violet Cell Proliferation Kit	Thermo Fisher Scientific	Cat# 2161821
Phusion™ High-Fidelity DNA Polymerase	Thermo Fisher Scientific	Cat# F530S

**Critical commercial assays**		

Cell Conditioning 1 (CC1)	Ventana, Roche Diagnostics	Cat# 950-224
UltraView Universal DAB Detection Kit	Ventana, Roche Diagnostics	Cat# 760-500
Ventana Benchmark ULTRA automated staining platform	Ventana, Roche Diagnostics	N/A
SuperScript™ IV Reverse Transcriptase	Invitrogen	Cat# 18090050
Random Hexamer Primers	Invitrogen	Cat# SO142
LightCycler 480 SYBR Green I Master Mix	Roche	Cat# 04887352001

**Deposited data**		

Pre-processed bulk RNA-seq data from pNET clinical samples and cell lines (this study)	Mendeley Data	https://data.mendeley.com/datasets/r9m66rjtxy/1
Raw single-nucleus RNA-seq count matrices and cell metadata (this study)	Mendeley Data	https://data.mendeley.com/datasets/r9m66rjtxy/1
Clinical patient sample metadata (this study)	This study	Table S2
RNA-seq profiles of TCGA-PAAD dataset	NCI Genomic Data Commons^46^	https://portal.gdc.cancer.gov/projects/TCGA-PAAD
Clinicopathological data of TCGA-PAAD dataset	cBioPortal^47^	https://www.cbioportal.org/
Whole genome sequencing profiles of Scarpa et al. dataset	Scarpa et al.^5^	https://ega-archive.org/datasets/EGAD00001002684
Whole genome sequencing profiles of Williamson et al. dataset	Williamson et al.^6^	https://www.bcgsc.ca/downloads/genomes/9606/hg19/1000genomes/bwa_ind/genome/
RNA-seq profiles of Chan et al. dataset	Chan et al.^7^	https://www.ncbi.nlm.nih.gov/geo/query/acc.cgi?acc=GSE118014
RNA-seq profiles of Alvarez et al. dataset	Alvarez et al.^18^	https://www.ncbi.nlm.nih.gov/geo/query/acc.cgi?acc=GSE98894

**Software and algorithms**		

fastp (v0.23.1)	Chen et al.^48^	https://github.com/OpenGene/fastp
STAR (v2.7.10b)	Dobin et al.^49^	https://github.com/alexdobin/STAR
featureCounts (v1.6.2)	Liao et al.^50^	https://subread.sourceforge.net/featureCounts.html
Cutadapt (v4.4)	Martin et al.^51^	https://cutadapt.readthedocs.io/en/v4.4/
STAR-Fusion (v1.13.0)	Haas et al.^52^	https://github.com/STAR-Fusion/STAR-Fusion
AccuFusion (in-house)	Su et al.^53^	https://pmc.ncbi.nlm.nih.gov/articles/PMC10429329/
R (v4.2.2)	R Core Team, 2022	https://www.r-project.org/
MCPcounter (v1.2.0)	Becht et al.^54^	https://github.com/ebecht/MCPcounter
estimate (v1.0.13)	Yoshihara et al.^55^	https://bioinformatics.mdanderson.org/estimate/rpackage.html
GSVA (v1.46.0)	Barbie et al.^56^	https://github.com/rcastelo/GSVA
limma (v3.54.0)	Ritchie et al.^57^	https://www.bioconductor.org/packages/limma/
MOVICS (v0.99.17)	Lu et al.^58^	https://github.com/xlucpu/MOVICS
clusterProfiler (v4.6.0)	Wu et al.^59^	https://bioconductor.org/packages/clusterProfiler/
GseaVis (v0.1.1)	Zhang et al.^60^	https://github.com/junjunlab/GseaVis
Nearest Template Prediction (NTP)	Hoshida et al.^61^	https://github.com/peterawe/CMScaller
pRRophetic (v0.5)	Geeleher et al.^62^	https://github.com/paulgeeleher/pRRophetic
CeleScope™ (v1.10.0)	Singleron Biotechnologies	www.github.com/singleron-RD/CeleScope
CellBender (v0.3.2)	Fleming et al.^63^	https://github.com/broadinstitute/CellBender
Scrublet (v0.2.3)	Wolock et al.^64^	https://github.com/swolock/scrublet
Seurat (v5.0.2)	Hao et al.^65^	https://github.com/satijalab/seurat
sctransform (v0.4.1)	Choudhary et al.^66^	https://github.com/satijalab/sctransform
SingleR (v2.0.0)	Aran et al.^67^	https://github.com/dviraran/SingleR
pySCENIC (v0.12.0)	Aibar et al.^68^	https://github.com/aertslab/pySCENIC
CellChat (v2.1.2)	Jin et al.^69^	https://github.com/jinworks/CellChat
Cytoscape (v3.9.1)	Shannon et al.^70^	https://cytoscape.org/
Connectivity Map (CMAP)	Subramanian et al.^71^	https://clue.io/
ggplot2 (v3.5.0)	CRAN	https://cran.r-project.org/web/packages/ggplot2/index.html
survival (v3.4.0)	CRAN	https://cran.r-project.org/web/packages/survival/index.html
survminer (v0.4.9)	CRAN	https://cran.r-project.org/web/packages/survminer/index.html
ClassDiscovery (v3.4.0)	CRAN	https://cran.r-project.org/web/packages/ClassDiscovery/index.html
harmony (v1.2.0)	CRAN	https://cran.r-project.org/web/packages/harmony/index.html
DWLS (v0.1.0)	CRAN	https://cran.r-project.org/web/packages/DWLS/index.html
ConsensusClusterPlus (v1.62.0)	Bioconductor	https://bioconductor.org/packages/devel/bioc/html/ConsensusClusterPlus.html
M3C (v1.20.0)	Bioconductor	https://www.bioconductor.org/packages/release/bioc/html/M3C.html
sva (v3.46.0)	Bioconductor	https://bioconductor.org/packages/devel/bioc/html/sva.html
ComplexHeatmap (v2.13.4)	Bioconductor	https://www.bioconductor.org/packages/release/bioc/html/ComplexHeatmap.html

**Other**		

Ensembl (Release 109)	Dyer et al.^72^	https://www.ensembl.org/Homo_sapiens/Info/Index
The Molecular Signatures Database (MSigDB)	Liberzon et al.^73^	https://www.gsea-msigdb.org/gsea/msigdb
PanglaoDB	Franzén et al.^74^	https://panglaodb.se/
Genomics of Drug Sensitivity in Cancer (GDSC)	Yang et al.^75^	https://www.cancerrxgene.org/
FigureYa	Lu et al.^76^	https://github.com/xlucpu/FigureYa

### Experimental model and study participant details

#### Sample collection and cohort description

We analyzed a subset of 74 FF primary pNET samples collected at the Pathology Department of CHU Strasbourg, derived from a previously described monocentric cohort of 187 patients.^12^ These 74 samples were selected based on the availability of high-quality FF material and were subjected to RNA-seq and IHC for BEND2 expression. No specific exclusion criteria were applied. The cohort included 30 females (40.5%) and 44 males (59.5%), with a median age of 57 years (range: 21–80 years). No patients had known immunodeficiency conditions at the time of tissue collection. A validation cohort of 13 FFPE primary pNET samples from the same patient cohort was additionally used for IHC validation (5 females, 8 males; median age 66 years, range: 41–85 years). The majority of tumor samples in our study (68/74, 92%) were obtained from treatment-naïve patients at the time of surgical resection. Six cases (8%) had received prior therapy: four patients were treated with somatostatin analogs (SSTa) for ≤3 months, while two patients were more heavily pretreated—one with SSTa, everolimus, and capecitabine chemotherapy, and another with SSTa combined with temozolomide plus capecitabine (Table S2). FF tissue was collected immediately after resection, and FFPE blocks were prepared according to standardized pathology workflows. Tissue selection and histological review were performed independently by two board-certified pathologists (M.-P.C. and A.F.), who ensured sampling of tumor-rich regions while avoiding necrotic or normal pancreatic tissue. Diagnosis, WHO classification, and tumor grade were confirmed for all cases. The study was approved by the local ethics committee, and all patients provided written informed consent for the use of their biological material in research.

#### The Cancer Genome Atlas pancreatic cancer cohort

The TCGA-PAAD dataset (*n* = 159) included 152 pancreatic ductal adenocarcinomas and seven misclassified pNETs, as reported previously.^77^ Clinicopathological features and survival data were obtained from cBioPortal.^46^

#### External validation cohort of pNET

Two publicly available pNET transcriptome datasets were included for validation: (i) 33 well-differentiated pNETs with annotated *ATRX/DAXX/MEN1* mutational status (GSE118014, Chan et al.^7^); and (ii) 83 primary pNETs (GSE98894, Alvarez et al.^18^).

#### Cell culture

The following human cell lines were utilized in the study and cultured under the specified conditions: BON1 (CVCL_3985) were supplemented with RPMI 1640 w/o HEPES (R-6504), 10% FCS Heat-Inactivated (35-079-CV), 100 UI/mL Penicillin-100 μg/mL Streptomycin (ref. 15140-130); and QGP1(CVCL_3143) were supplemented with RPMI 1640 w/o HEPES (R-6504) + 10.0% Tet Free Fetal Calf Serum (Dutscher) (35-079-CV) + PS 1% = Penicilline 100 UI/mL - Streptomycine 100 μg/mL (15140-130).

### Method details

#### Tissue processing and histopathological evaluation

All surgical specimens were oriented and inked in the operating room by the surgeon according to a previously described protocol.^78^ Macroscopic handling was standardized throughout the study. Tumor description and biobank conservation were performed by the pathologist immediately upon receipt of the specimen, prior to fixation in 10% buffered formalin for 16 h at room temperature. When necessary, re-inking of resection margins was performed after fixation, and macroscopic three-dimensional tumor size was measured. Pancreatic tumor specimens, together with peripancreatic lymph nodes resected *en bloc* with the pancreatic specimen (including spleno-mesenterico-portal nodes), were then axially sectioned into 5–7 mm slices, as proposed by Verbeke et al.^79^ For pancreaticoduodenectomy specimens, all slices were entirely processed using a large-section histopathology protocol originally introduced for breast tumors.^80^ Slices were embedded in mega-cassettes (50 × 35 × 20 mm or larger) and processed in a TissueTek VIP6AI with a 21 h program, followed by 24 h incubation in histowax at 60°C. The slices were embedded in paraffin blocks (70 × 50 × 15 mm or 110 × 65 × 25 mm) and sectioned using a Microm-Thermo Scientific HM340E microtome. At least two levels at 500 μm and 1,000 μm were cut from each block, expanded on a heating plate at 44°C over a 2.4% glycerol–albumin solution, mounted on large glass slides (135 × 85 mm), and H&E-stained using a TissueTek DRS 2000. For splenopancreatectomy specimens, all axial cuts were processed in standard paraffin blocks.

#### Immunohistochemistry analysis

FFPE tissue blocks from patients with BEND2 alterations in the CHU Strasbourg cohort were selected for IHC. For each case, the most representative tumor block was retrieved, and 5-μm sections were cut and stained using a rabbit polyclonal anti-BEND2 antibody (anti-CXorf20, Abcam #ab204795; dilution 1:50) on the Ventana Benchmark ULTRA platform (Ventana Medical Systems, Roche Tissue Diagnostics, Tucson, AZ, USA). Antigen retrieval was performed using Cell Conditioning 1 (CC1, Ventana) for 64 min at 95°C. Slides were then incubated with the primary antibody for 32 min at room temperature, followed by detection with the ultraView Universal DAB detection kit. Hematoxylin was used for counterstaining. To validate the specificity of the antibody, we constructed a TMA comprising 74 pNET cases with available RNA-seq data. Only tumors harboring *BEND2* fusions exhibited positive BEND2 protein expression, confirming antibody specificity. For further validation, 13 independent pNET FFPE samples were analyzed using the same IHC protocol and section thickness (5 μm). Additional IHC assays for CDX2 and gastrin (GAST) were performed on TMA sections using the same Ventana Benchmark ULTRA platform in the Department of Pathology at the University Hospital of Strasbourg, where these markers are routinely assessed. Antigen retrieval was conducted using CC1 buffer for 36 min (CDX2 and gastrin). Slides were incubated with the following primary antibodies at room temperature: CDX2 (Zytomed, clone EPR2764Y, 1:100) for 32 min, and gastrin (Cell Marque, polyclonal, 1:50) for 20 min. Detection and counterstaining steps were performed as described above. All slides were evaluated by a board-certified pathologist (A.F.). Staining was scored as positive when nuclear staining was observed for CDX2 or cytoplasmic staining for gastrin. The H-score was calculated for each tumor by multiplying the percentage of positively stained tumor cells by the staining intensity (0–3), yielding a semi-quantitative measure of marker expression.

#### Nucleic acid extraction

RNA extraction from human tumor samples was performed using the AllPrep DNA/RNA Mini Kit (Qiagen) according to the manufacturer’s instructions. Quality control of extracted nucleic acids was done using an Agilent 2100 Bioanalyzer. The cell lysates were collected for RNA extraction using a standard TRIzol RNA extraction, in accordance with the manufacturer’s instructions.

#### RNA sequencing

Total RNA for 74 pNET samples was converted in sequencing libraries using the NEBNext UltraTM RNA Library Prep Kit for Illumina (NEB, USA) following the manufacturer’s recommendations. To select cDNA fragments of preferentially 150–200 bp in length, the library fragments were purified with the AMPure XP system (Beckman Coulter, Beverly, USA). Then 3 μL USER Enzyme (NEB, USA) was used with size-selected, adaptor-ligated cDNA at 37°C for 15 min followed by 5 min at 95°C before PCR. Then PCR was performed with Phusion High-Fidelity DNA polymerase, Universal PCR primers and Index (X) Primer. At last, PCR products were purified (AMPure XP system) and library quality was assessed on the Agilent Bioanalyzer 2100 system. The library preparations were sequenced on an Illumina platform and paired-end reads were generated. Raw FASTQ reads were quality-filtered and trimmed using fastp (v0.23.1). The GRCh38 reference genome and corresponding gene annotation (Ensembl release 109) were downloaded from Ensembl. Clean paired-end reads were aligned to the GRCh38/hg38 human reference genome using STAR (v2.7.10b), and read counts per gene were obtained with FeatureCounts (v1.6.2).^50^ Expression levels were calculated as fragments per kilobase of exon per million mapped reads (FPKM) and subsequently converted to transcripts per kilobase million (TPM).

#### Fusion detection

We used STAR-Fusion (v1.13.0) to identify fusion genes in tumor samples.^52^ By enabling the STAR option to report the chimeric read alignments, these data will be readily available for running STAR-Fusion, quickly providing access to lists of candidate fusion transcripts. Those fusions that pass the filters are reported in a tab-delimited summary file identifying the fusion pairs, the inferred fusion breakpoint (chromosomal exon boundaries), counts of supporting split reads and spanning fragments, and identification of the RNA-Seq reads that support the fusion prediction. The parameters of “--min_junction_reads” and “--min_sum_frags” were assigned default values. To validate the predicted fusions, we also employed AccuFusion, an in-house tool recently developed by our team.^53^

#### Processing of TCGA-PAAD data

Raw FASTQ files for the TCGA-PAAD project were downloaded from the Genomic Data Commons. Reads were aligned to the GRCh38/hg38 reference genome using the same pipeline as for our in-house cohort, and raw counts were converted to TPM values. Batch effects between pNET and PAAD samples were adjusted using an empirical Bayes framework through R package sva (v3.46.0),^81^ and evaluated via PCA.

#### Processing of external pNET validation datasets

Expression data from GSE118014 and GSE98894 were processed as log_2_-transformed TPM values. For this study, cases with *BEND2* alterations were defined as those with TPM values >1.

#### Single nuclei isolation

The single nuclei suspension was obtained using GEXSCOPE Single Nucleus RNA Library Kit V2 (Singleron Biotechnologies). Briefly, on ice the tissue was immersed in cold nucleus separation solution (Singleron Biotechnologies) and cut into small pieces. Further homogenization was achieved by performing 5 strokes with pestle A and 5 strokes with pestle B of the Kimble douncer (KIMBLE KONTES Dounce Tissue Grinder, cat. nr. 885300-0002). The sample was then incubated on ice for 15 min where the state of dissociation was monitored every 5 min under a light microscope. Following homogenization and digestion, the suspension was filtered using a 40-μm sterile strainer (Greiner, cat. nr. 542040). The nuclei suspension was centrifuged at 200xg for 2 min at 4°C, and the supernatant was centrifuged at 500xg for 5 min at 4°C. The resulting pellet containing nuclei was resuspended in 0.25 mL of cold nuclei suspension buffer (Singleron Biotechnologies). The quality of the nuclei was assessed by Trypan Blue staining (0.4% w/v, Gibco) under a light microscope. The nuclei were counted using propidium iodide with a Luna FX7 automated cell counter (Logos Biosystems, Villeneuve d’Ascq, France).

#### Single-nuclei RNA sequencing library preparation

A total of 30,000 nuclei were loaded onto a microfluidic chip (Singleron GEXSCOPE Single Nucleus RNA Library Kit V2) to ensure the loading of 6000 nuclei. The single nucleus RNA-seq libraries were constructed using (GEXSCOPE Single Nucleus RNAseq Library Kit, Singleron Biotechnologies) according to manufacturer's instructions. Paramagnetic beads conjugated to oligodT probes that carry a UMI and a barcode unique to each bead (from the same kit) were loaded, after which the nuclei were lysed. The beads bound to polyadenylated mRNA were extracted from the chip and reverse transcribed into cDNA at 42°C for 1.5 h, and the cDNA amplified by PCR. The cDNA was then fragmented and ligated to indexed Illumina adapters. The fragment size distribution of the final amplified library was obtained on an Agilent Fragment Analyzer.

#### Library sequencing

The library concentration was calculated using the Qubit 4.0 fluorometer and the libraries were pooled in an equimolar fashion. The single nucleus libraries were sequenced on an Illumina NovaSeq 6000 using a 2 × 150-bp approach to a final depth of 45 GB per library. The reads were demultiplexed according to the multiplexing index sequencing on Illumina’s BaseCloud platform.

#### Raw data processing and quality control

CeleScope (v1.10.0) was used to process the raw data, demultiplex cellular barcodes, map reads to the transcriptome of the human reference genome GRCh38, and downsample reads (www.github.com/singleron-RD/CeleScope; Singleron Biotechnologies). Briefly, fastq files were demultiplexed according to their respective cell barcodes and UMIs. Adapter sequences and poly A tails were trimmed by Cutadapt (v4.4) and the trimmed Read2 reads were aligned to the GRCh38 version of the human genome using STAR (2.7.10b) with Ensembl version 109 gene annotations by featureCounts (v1.6.2).^49^^,^^50^^,^^51^ Reads with the same cell barcode, UMI and gene were grouped together to calculate the number of UMIs per gene per cell. The UMI count tables of each cellular barcode were merged as a raw UMI count matrix, which was converted into a Seurat object using the R package Seurat (v4.1.0).^65^ To eliminate empty droplets and technical artifacts, we applied CellBender (v0.3.2) and detected and removed doublets with Scrublet (v0.2.3).^63^^,^^64^ Quality assessment of nuclei was based on three metrics: (1) library size between 1,000 and 10,000 total UMI counts per nucleus, (2) detection of more than 500 genes, and (3) less than 10% mitochondrial gene expression. After quality control filtering, we obtained 43,619 single nuclei that were used for downstream analyses.

#### Data integration and the dimensionality reduction

After data processing, the Seurat object with gene expression data from individual samples was created using the Read10X() function, and the data were merged together. SCTransfrom was then used to normalize and scale the expression data, with cell-cycle effects accounted for by a regression-based approach using the CellCycleScoring() function in Seurat, and the top 3,000 highly variable genes (HVGs) were identified.^82^ After that we performed the PCA based on these HVGs. The batch effects were removed by the “harmony” R package (v1.2.0), in which the samples are treated as the batch. The number of components for downstream analysis was selected when the cumulative standard deviations were larger than 100 for the first time.

#### Cell clustering and annotation

The clustering analysis was performed based on the integrated joint embedding produced by Harmony after computing a shared nearest-neighbor graph using the FindNeighbors(k.param = 30) function with the Louvain algorithm (resolution = 0.25), which was implanted in the “FindClusters” function of the Seurat package. The identified clusters were visualized on the 2D map produced with the uniform manifold approximation and projection (UMAP) method. For sub-clustering analysis, a similar procedure was followed, including the identification of variable genes, dimension reduction, cell integration with Harmony, and clustering identification, but this time focused on the restricted cluster derived from the overall analysis. To annotate the cell clusters, differentially expressed genes (DEGs) with high discrimination abilities between the groups were identified with the FindAllMarkers() function in Seurat using the default non-parametric Wilcoxon rank-sum test with Bonferroni correction. The cell groups were assigned via the SingleR package (v2.0.0),^67^ using annotated data from mouse pancreatic cells as ref.^83^

#### Cell classification using bulk-signature

The bulk RNA-seq signature was used to score each cell in the snRNA-seq dataset using the AddModuleScore() function from Seurat, which allows for the scoring of individual cells based on a pre-defined gene signature. Each cell was assigned a putative label based on the highest-scoring signature.

#### Enrichment analysis of snRNA-seq data

The non-parametric and unsupervised algorithm GSVA was applied to assess the relative pathway activities in tumor cells based on the 50 Hallmark pathways.

#### Gene regulatory network inference

Single cell regulatory network inference and clustering (SCENIC) was employed to infer TF networks active in snRNA-seq data of pNET. Analysis was performed using pySCENIC (v0.12.0) with recommended parameters,^68^ which analyzed the co-expression of transcriptional factors and their putative target genes. Potential regulons based on DNA-motif analysis were selected by hg38 RcisTarget, and active gene networks were identified by AUCell. To determine essential regulators of each bulk classification using snRNA-seq data, the Regulon Specificity Score (RSS) was calculated by reimplementing pySCENIC’s regulon_specificity_scores function in R and plotted as a rank ordered scatterplot. The specificity score measures the extent to which a transcription factor regulates a particular gene compared to other genes within the regulon. A high specificity score indicates that the transcription factor has a strong regulatory effect on a particular gene, while a low specificity score indicates that the transcription factor has a weaker effect on that gene.

#### Cell–cell communication

Cell–cell interactions based on the expression of known L–R pairs in different cell types were calculated using CellChat (v2.1.2).^69^ In brief, for each tumor subtype with TME components the gene expression data of nuclei and assigned cell type were used as input for CellChat. First, overexpressed ligands or receptors in one cell group were identified, the overexpressed L–R interactions were identified if either the ligand or receptor was overexpressed. Next, CellChat was used to infer the biologically significant cell–cell communication by assigning each interaction a probability and performing a permutation test. Interactions with probability that hit at the lower quartile were considered unlikely to be a robust connection and they were subsequently discarded. Communication networks were visualized by Cytoscape (v3.9.1) and interaction probability visualized using a bubble plot.^70^

#### Deconvolution of cell-type compositions in bulk RNA-seq data

Cell-type compositions from bulk RNA-seq data were estimated by deconvolution from single-nuclei data using the dampened weighted least squares (DWLS) algorithm through the R package DWLS (v0.1.0), which corrects common biases toward cell types that are characterized by highly expressed genes and/or are highly prevalent, further providing accurate detection across diverse cell types.^84^

#### Connectivity Map analysis and in-silico drug sensitivity prediction

CMap contains over 7,000 gene expression profiles representing ∼1,300 compounds and enables the identification of potential therapeutic agents by linking gene expression signatures to drug perturbations. Compounds with connectivity scores ≤ −99.5 were considered as candidates with potential therapeutic effects.^71^^,^^85^ To further evaluate the therapeutic relevance, we applied pRRophetic (v0.5) to predict drug sensitivity for each pNET sample, using expression profiles from tumors as the testing set and drug sensitivity data from the GDSC database as the training set, as previously described.^62^^,^^86^ The GDSC dataset includes both transcriptomic profiles and half maximal inhibitory concentration (IC_50_) values for 727 human cancer cell lines, where lower IC_50_ values indicate greater drug sensitivity.^75^ Missing IC_50_ values were imputed using k-nearest neighbor imputation. Predicted IC_50_ values for each pNET sample and drug were obtained by ridge regression, and model accuracy was assessed by 10-fold cross-validation within the GDSC training set.

#### Plasmid cloning and lentiviral transduction

The plasmids pLenti-TetON-FLAG-HA-mCherry, pLenti-TetON-FLAG-HA-*BEND2*, pLenti-TetON-FLAG-HA-*EWSR1*, pLenti-TetON-FLAG-HA-*CHD7*-*BEND2*, and pLenti-TetON-FLAG-HA-*EWSR1*-*BEND2* were synthesized by the IGBMC platform. Gene expression was driven by a TRE promoter Plasmids were transiently transfected into HEK293T cells using PEI (Polysciences, ref. 23966). Lentiviral particles were produced in HEK293T, purified via ultracentrifugation, and resuspended in PBS. Target cell lines were transduced with the respective viruses and selected using puromycin (1 μg/mL). Stable overexpression of *BEND2, EWSR1*, *CHD7-BEND2*, and *EWSR1-BEND2* was confirmed by RT-qPCR and western blotting using Rabbit Polyclonal CXorf20 (BEND2) (Abcam, ab204795) antibody, and further validated by RNA-seq.

#### Lentivirus production

Lentivirus production was performed using the HEK293T packaging cell line. The 10 cm plate was treated with poly D lysine and later Cells were seeded in achieve approximately 70–80% confluency at the time of transfection. Lentiviral particles were generated by co-transfecting the cells with a transfer plasmid containing the gene of interest, a packaging plasmid, and an envelope plasmid using Lipofectamine 2000 reagent. A total of 14 μg of plasmid DNA, consisting of 7 μg of the transfer plasmid encoding the gene of interest, 4.5 μg of the packaging plasmid, and 2.5 μg of the envelope plasmid, was diluted in 500 μL of Opti-MEM. Separately, 30 μL of Lipofectamine 2000 Transfection Reagent (Invitrogen; ref. 11668027) was diluted in 500 μL of Opti-MEM (ref. 31985062) and incubated for 5 min at room temperature. The DNA and Lipofectamine 2000 mixtures were combined and incubated for 20 min to form DNA-lipid complexes. This transfection mixture was added dropwise to HEK293T cells in a 10 cm dish, ensuring even distribution. After 4–6 h, the transfection medium was replaced with fresh DMEM containing 10% FBS. Viral supernatants were collected 48 h post-transfection, centrifuged at 300 × g for 5 min to remove cellular debris, and filtered through a 0.45 μm syringe filter. Aliquots of the viral preparation were stored at −80°C for further use.

#### Lentivirus infection

The cells of interest were infected with lentiviral particles to achieve stable gene expression. Cells were seeded in a 6-well plate at a density to have 50–70% confluency on the day of infection. Lentiviral particles were thawed on ice and diluted in cell specific media containing 10% FBS. Polybrene was added to the virus-containing medium at a final concentration of 8 μg/mL to enhance infection efficiency. The culture medium was removed from the target cells, and 1–2 mL of the virus-containing medium was added per well. Plates were gently swirled to ensure even distribution and incubated at 37°C with 5% CO_2_ for 8–16 h. After incubation, the viral medium was replaced with fresh media containing 10% FBS. For selection of stably transduced cells, puromycine was added 48h post-infection, with the medium being replaced every day for 7 days until all non-transduced cells were eliminated. Transduction efficiency was assessed using by performing qPCR or the western blot against gene of interest.

#### Reverse transcription-quantitative polymerase chain reaction

Reverse transcription-quantitative polymerase chain reaction (RT-qPCR) was conducted using SuperScript IV Reverse Transcriptase (Invitrogen, 18090050) with random hexamer primers (Invitrogen, SO142). The RT-qPCR was conducted in a 20-μL reaction system utilizing a LightCycler-480 SYBR master mix (Roche, 04887352001) in accordance with the manufacturer’s instructions. The relative expression of the genes of interest were normalized according to housekeeping 18S by calculating the standard 2−ΔΔCt method. There are 3 biological experiments, and each biological experiment was conducted in triplicate.

**Table:** Primers

Name	Sequence
*BEND2_exon_4–5_F*	AGCCCATGGTGACCAAATAG
*BEND2_exon_4–5_R*	GCAGTTCATGACATGCTGCT
*EWSR1_exon_5–6_F*	GCCTCCTATGCAGCTCAGTC
*EWSR1_exon_5–6_R*	GGTTGTAACCCCCTGTGCTA
*EWSR_BEND2_junction_F*	ATCCTACAGCCAAGCTCCAA
*EWSR_BEND2_junction_R*	AGAATCCTGGCCACTGTCAC
*CHD7_BEND2_junction_F*	CTTCACCTCCACACCCTCAT
*CHD7_BEND2_junction_R*	GCAGTTCATGACATGCTGCT
*18S_F*	CACGGACAGGATTGACAGATTG
*18S_R*	CACGGACAGGATTGACAGATTG

#### Western blot

The BON1 cell line over expressing *BEND2* (B), *EWSR1* (E), *CHD7*-*BEND2* (CB), *EWSR1*-*BEND2* (EB) and mCherry (M) were plated in dish. After 24h of plating these cells were induced with doxycycline. The cells were collected after 48-h of doxycycline induction. They were subjected to a washing process using phosphate-buffered saline (PBS, 1×). After washing the cells were detached from the surface of the plate in a tube and subjected to centrifugation, after which the supernatant was discarded. The pellet was suspended in LSDB buffer (2×) and 1× Protease Inhibitor (#40091500, Sigma-Aldrich), and subsequently maintained in liquid nitrogen for a period of 2 min. Subsequently, the sample was transferred to a water bath maintained at 37°C for a period of 2 min. The incubation steps were repeated twice, followed by centrifugation at 4°C. Next, the supernatant was transferred to a separate tube. The total protein concentration was determined by dissolving 1 μL of the total protein extract in protein assay dye (1×) (#5000006, Bio-Rad) and quantifying the amount by the Bradford dye-binding method. The protein samples were subjected to electrophoresis on a NuPAGE gel (4–12% bis-tris gel, #20070610, Invitrogen) and subsequently transferred onto a polyvinylidene fluoride (PVDF) membrane. The membrane was initially blocked using 5% bovine serum albumin (BSA), followed by overnight incubation with the primary antibody. On the following day, the membrane was rinsed with PBST (phosphate-buffered saline, 0.01% Tween 20) and later blocked with the secondary antibody for 1h. Later, the membrane was rinsed with PBST, and the blot was treated with ECL reagent (UC180107, Protein Biology) for signal acquisition using the imaging system. The primary antibodies utilized were Rabbit Polyclonal CXorf20 (BEND2) (Abcam, ab204795) with 1:100 dilution and actin with dilution 1:5000.

#### Anchorage-dependent assay

The BON1 cell lines, transfected with *BEND2* (B), *EWSR1* (E), *CHD7-BEND2* (CB), *EWSR1-BEND2* (EB) and *mCherry* (M) plasmids were seeded at a density of 1000 per well in six-well plates and were maintained at 37°C with 5% CO_2_ supplementation. The doxycycline was added to media after every 48h. The cells were incubated for a period of 14 days (BON1) in the culture environment. At the end of the assay, the media from the cells was removed, washed with cold PBS (1×), and fixed with 3.4% formaldehyde. The colonies were subsequently stained with crystal violet. Later, images were captured, and the colonies were quantified using the ImageJ software (1.53n). There are more than 3 biological experiments, and each biological experiment was conducted in triplicate.

#### Cell proliferation assay

Cell proliferation was determined using Invitrogen CellTrace (#2161821) according to the manufacturer’s instructions. The BON1 cell lines over expressing *BEND2* (B), *EWSR1* (E), *CHD7-BEND2* (CB), *EWSR1-BEND2* (EB) and *mCherry* (M) control were plated in a 6-well plate. The next day, these cells were treated with Cell Trace Violet dye for 15 min. The cells were washed with media and the dye was replaced with the fresh media containing doxycycline. After 72 h of incubation, the cells were trypsinized and centrifuged. The pellet was suspended in PBS (1×) and the cells were used to analyze by flow cytometry (BD. FORTRESSA X20). The data obtained were analyzed using FlowJob software. There are more than three independent biological experiments performed, and each biological experiment was conducted in triplicate.

#### Agarose gel electrophoresis

RNA (500 ng) from four human tumor samples and matched healthy tissue was reverse-transcribed using SuperScript IV Reverse Transcriptase (Invitrogen, Cat#18090050) with random hexamer primers (Invitrogen, Cat#SO142), following the manufacturer’s instructions. PCR amplification was performed on 10 ng of cDNA with primers designed to span the fusion junctions, using Phusion High-Fidelity DNA Polymerase (Thermo Fisher Scientific, Cat#F530S). For CHD7-BEND2, the forward primer targeted CHD7 exon 1 and the reverse primer targeted BEND2 exon 5. For EWSR1-BEND2, the forward primer targeted EWSR1 exon 7 and the reverse primer targeted BEND2 exon 9. Expected PCR product sizes were 166 bp for EWSR1-BEND2 and 186 bp for CHD7-BEND2. PCR products were separated by agarose gel electrophoresis on a 2% gel containing ethidium bromide, run at 130 V for 40 min in TAE buffer, and visualized under UV illumination.

### Quantification and statistical analysis

#### Consensus clustering

To reduce noise and enhance clustering robustness, we filtered out low expressed mRNA whose TPM value was equal to zero in at least 10% of the samples. Only primary tumor samples (*n* = 75; 74 FF CHU Strasbourg and 1 TCGA) were included in this analysis; no metastatic tissue was sequenced. Thus, the selected genes reflect the transcriptomic variability of primary tumors. Those mRNAs with the highest variability measured by median absolute deviation were selected and the corresponding gene expression data was log_2_ transformed prior to clustering. Unsupervised consensus hierarchical clustering was then performed by using R package ConsensusClusterPlus (v.1.62.0).^87^ To be specific, the consensus process was set to 90% of features re-sampling with 1,000 perturbations. Specifically, Ward’s clustering method and 1-Pearson’s coefficient as distance measures were used for each perturbation. The final hierarchical clustering based on the consensus matrix used 1-Pearson’s correlation as distance with Ward’s clustering method. The optimal clustering number was estimated by calculating the stability index using Monte Carlo reference-based consensus clustering through R package M3C (v1.20.0).^88^

#### Differential and enrichment analyses

Differential expression analyses were conducted using the “one vs. others” mode through the R package MOVICS (v0.99.17).^58^^,^^89^ For GSEA, a pre-ranked gene list was generated based on log_2_ fold-change values from the differential expression results. Functional enrichment was then assessed using the clusterProfiler package (v4.6.0) and visualized by GseaVis package (v0.1.1), with gene sets sourced from the the Molecular Signatures Database (MSigDB).^59^^,^^60^

#### Abundance estimation of tumor microenvironment

The population abundance of tissue-infiltrating immune and stromal cell populations were estimated by R package MCPcounter (v1.2.0) per sample using normalized count data.^54^^,^^90^ The presence of infiltrating immune/stromal cells and tumor purity in tumor tissue was estimated by R package “estimate” (1.0.13).^55^

#### Cell identity analyses

To study cell identity from a transcriptomic aspect, we extracted a list of gene markers for nine pancreatic cell types, including acinar, alpha, beta, delta, ductal, epsilon, gamma (PP), pancreatic progenitor, and pancreatic stellate cells, using PanglaoDB.^74^ Individual enrichment for each of the cell types was quantified using Gene Set Variation Analysis (GSVA).

#### Statistical analyses

All statistical tests were executed by R (v4.2.2), including Fisher’s exact test for categorical data, a two-sample Mann-Whitney U test for continuous data, and Spearman’s coefficient for correlation analysis. Survival rates were analyzed using Kaplan-Meier curves using R package survival (v3.4.0), with differences determined using a log rank test. Hazard ratios and 95% CI were calculated using Cox proportional hazard regression. Relationships between genes of interest and patient survival were computed using R package survminer (v0.4.9) where patients were stratified using an optimal cutoff determined by the maximally selected rank statistics.^91^ Propensity score matching was not performed because the primary aim of this study was to discover and characterize molecular subtypes rather than to estimate causal effects, and the rarity of certain subtypes would have further reduced sample sizes and statistical power. For unadjusted comparisons, a *p* < 0.05 was considered statistically significant.

## Supplemental information

Document S1. Figures S1–S17

Table S1. Clinical characteristics of pNET datasets used for BEND2 fusion analysis and molecular subtype validation, related to Figure 1

Table S2. Clinicopathological characteristics of the 75 pNET samples analyzed by RNA sequencing, related to Figure 1

Table S3. List of predicted fusion transcripts using STAR-fusions and validated by an independent algorithm, related to Figure 1 and STAR Methods

Table S4. Genetic details of five BEND2 fusions in pNETs detected from RNA-seq, related to Figure 2 and STAR Methods

Table S5. Clinicopathological characteristics of the 13 FFPE pNET samples, related to Figure 2

Table S6. Summary of clinical characteristics stratified by BEND2 fusion status in 187 pNETs, related to Figure 2

Table S7. Differentially expressed gene list for five bulk subtypes, related to Figure S4

Table S8. Clinicopathological and molecular associations of the transcriptomic subtypes (*n* = 75), related to Figure 3

Table S9. 1,000-gene signature related to the five bulk pNETs subtypes, related to Figure S7 and STAR Methods

Table S10. Summary of quality control for nine snRNA-seq profiles, related to Figure 4 and STAR Methods

Table S11. Cell-type composition per sample in the single-nuclei RNA-seq dataset, related to Figure 4 and Figure S12

Table S12. Summary of univariate Cox proportional hazard regression using tumor cell proportion of pseudo-bulk sn-clusters, related to Figure S12

Table S13. Characteristics of human pNET cell lines and results of STAR-Fusion analysis, related to Figure 6 and STAR Methods

Document S2. Article plus supplemental information

## References

[1] Dasari A., Shen C., Halperin D., Zhao B., Zhou S., Xu Y., Shih T., Yao J.C. (2017). Trends in the Incidence, Prevalence, and Survival Outcomes in Patients With Neuroendocrine Tumors in the United States. JAMA Oncol..

[2] Falconi M., Eriksson B., Kaltsas G., Bartsch D.K., Capdevila J., Caplin M., Kos-Kudla B., Kwekkeboom D., Rindi G., Klöppel G. (2016). ENETS Consensus Guidelines Update for the Management of Patients with Functional Pancreatic Neuroendocrine Tumors and Non-Functional Pancreatic Neuroendocrine Tumors. Neuroendocrinology.

[3] Falconi M., Bartsch D.K., Eriksson B., Klöppel G., Lopes J.M., O'Connor J.M., Salazar R., Taal B.G., Vullierme M.P., O'Toole D., Barcelona Consensus Conference participants (2012). ENETS Consensus Guidelines for the management of patients with digestive neuroendocrine neoplasms of the digestive system: well-differentiated pancreatic non-functioning tumors. Neuroendocrinology.

[4] Rindi G., Mete O., Uccella S., Basturk O., La Rosa S., Brosens L.A.A., Ezzat S., de Herder W.W., Klimstra D.S., Papotti M., Asa S.L. (2022). Overview of the 2022 WHO Classification of Neuroendocrine Neoplasms. Endocr. Pathol..

[5] Scarpa A., Chang D.K., Nones K., Corbo V., Patch A.M., Bailey P., Lawlor R.T., Johns A.L., Miller D.K., Mafficini A. (2017). Whole-genome landscape of pancreatic neuroendocrine tumours. Nature.

[6] Williamson L.M., Steel M., Grewal J.K., Thibodeau M.L., Zhao E.Y., Loree J.M., Yang K.C., Gorski S.M., Mungall A.J., Mungall K.L. (2019). Genomic characterization of a well-differentiated grade 3 pancreatic neuroendocrine tumor. Cold Spring Harbor molecular case studies *5*. Cold Spring Harb. Mol. Case Stud..

[7] Chan C.S., Laddha S.V., Lewis P.W., Koletsky M.S., Robzyk K., Da Silva E., Torres P.J., Untch B.R., Li J., Bose P. (2018). ATRX, DAXX or MEN1 mutant pancreatic neuroendocrine tumors are a distinct alpha-cell signature subgroup. Nat. Commun..

[8] Cejas P., Drier Y., Dreijerink K.M.A., Brosens L.A.A., Deshpande V., Epstein C.B., Conemans E.B., Morsink F.H.M., Graham M.K., Valk G.D. (2019). Enhancer signatures stratify and predict outcomes of non-functional pancreatic neuroendocrine tumors. Nat. Med..

[9] Di Domenico A., Pipinikas C.P., Maire R.S., Bräutigam K., Simillion C., Dettmer M.S., Vassella E., Thirlwell C., Perren A., Marinoni I. (2020). Epigenetic landscape of pancreatic neuroendocrine tumours reveals distinct cells of origin and means of tumour progression. Commun. Biol..

[10] Yang K.C., Kalloger S.E., Aird J.J., Lee M.K.C., Rushton C., Mungall K.L., Mungall A.J., Gao D., Chow C., Xu J. (2021). Proteotranscriptomic classification and characterization of pancreatic neuroendocrine neoplasms. Cell Rep..

[11] Ji S., Cao L., Gao J., Du Y., Ye Z., Lou X., Liu F., Zhang Y., Xu J., Shi X. (2025). Proteogenomic characterization of non-functional pancreatic neuroendocrine tumors unravels clinically relevant subgroups. Cancer Cell.

[12] Debien V., Davidson G., Baltzinger P., Kurtz J.E., Séverac F., Imperiale A., Pessaux P., Addeo P., Bachellier P., Su X. (2021). Involvement of Neutrophils in Metastatic Evolution of Pancreatic Neuroendocrine Tumors. Cancers (Basel).

[13] Mafficini A., Scarpa A. (2018). Genomic landscape of pancreatic neuroendocrine tumours: the International Cancer Genome Consortium. J. Endocrinol..

[14] Muraro M.J., Dharmadhikari G., Grün D., Groen N., Dielen T., Jansen E., van Gurp L., Engelse M.A., de Koning E.J., van Oudenaarden A. (2016). A Single-Cell Transcriptome Atlas of the Human Pancreas. Cell Syst..

[15] Capelli P., Martignoni G., Pedica F., Falconi M., Antonello D., Malpeli G., Scarpa A. (2009). Endocrine neoplasms of the pancreas: pathologic and genetic features. Arch. Pathol. Lab Med..

[16] Kasajima A., Yazdani S., Sasano H. (2015). Pathology diagnosis of pancreatic neuroendocrine tumors. J. Hepatobiliary. Pancreat. Sci..

[17] Takahashi D., Kojima M., Suzuki T., Sugimoto M., Kobayashi S., Takahashi S., Konishi M., Gotohda N., Ikeda M., Nakatsura T. (2018). Profiling the Tumour Immune Microenvironment in Pancreatic Neuroendocrine Neoplasms with Multispectral Imaging Indicates Distinct Subpopulation Characteristics Concordant with whom 2017 Classification. Sci. Rep..

[18] Alvarez M.J., Subramaniam P.S., Tang L.H., Grunn A., Aburi M., Rieckhof G., Komissarova E.V., Hagan E.A., Bodei L., Clemons P.A. (2018). A precision oncology approach to the pharmacological targeting of mechanistic dependencies in neuroendocrine tumors. Nat. Genet..

[19] Ma Z., Zhang X., Zhong W., Yi H., Chen X., Zhao Y., Ma Y., Song E., Xu T. (2023). Deciphering early human pancreas development at the single-cell level. Nat. Commun..

[20] Miquelajáuregui A., Sandoval-Schaefer T., Martínez-Armenta M., Pérez-Martínez L., Cárabez A., Zhao Y., Heide M., Alvarez-Bolado G., Varela-Echavarría A. (2015). LIM homeobox protein 5 (Lhx5) is essential for mamillary body development. Front. Neuroanat..

[21] Tsuyama T., Sato Y., Yoshizawa T., Matsuoka T., Yamagata K. (2023). Hypoxia causes pancreatic β-cell dysfunction and impairs insulin secretion by activating the transcriptional repressor BHLHE40. EMBO. Rep..

[22] Xuan S., Sussel L. (2016). GATA4 and GATA6 regulate pancreatic endoderm identity through inhibition of hedgehog signaling. Development.

[23] Ireland A.S., Micinski A.M., Kastner D.W., Guo B., Wait S.J., Spainhower K.B., Conley C.C., Chen O.S., Guthrie M.R., Soltero D. (2020). MYC Drives Temporal Evolution of Small Cell Lung Cancer Subtypes by Reprogramming Neuroendocrine Fate. Cancer Cell.

[24] Gay C.M., Stewart C.A., Park E.M., Diao L., Groves S.M., Heeke S., Nabet B.Y., Fujimoto J., Solis L.M., Lu W. (2021). Patterns of transcription factor programs and immune pathway activation define four major subtypes of SCLC with distinct therapeutic vulnerabilities. Cancer Cell.

[25] Zhang W., Girard L., Zhang Y.A., Haruki T., Papari-Zareei M., Stastny V., Ghayee H.K., Pacak K., Oliver T.G., Minna J.D., Gazdar A.F. (2018). Small cell lung cancer tumors and preclinical models display heterogeneity of neuroendocrine phenotypes. Transl. Lung Cancer Res..

[26] Heslop J.A., Pournasr B., Liu J.T., Duncan S.A. (2021). GATA6 defines endoderm fate by controlling chromatin accessibility during differentiation of human-induced pluripotent stem cells. Cell Rep..

[27] Agaimy A., Kasajima A., Stoehr R., Haller F., Schubart C., Tögel L., Pfarr N., von Werder A., Pavel M.E., Sessa F. (2023). Gene fusions are frequent in ACTH-secreting neuroendocrine neoplasms of the pancreas, but not in their non-pancreatic counterparts. Virchows Arch..

[28] Sturm D., Orr B.A., Toprak U.H., Hovestadt V., Jones D.T.W., Capper D., Sill M., Buchhalter I., Northcott P.A., Leis I. (2016). New Brain Tumor Entities Emerge from Molecular Classification of CNS-PNETs. Cell.

[29] Lucas C.H.G., Gupta R., Wu J., Shah K., Ravindranathan A., Barreto J., Gener M., Ginn K.F., Prall O.W.J., Xu H. (2022). EWSR1-BEND2 fusion defines an epigenetically distinct subtype of astroblastoma. Acta Neuropathol..

[30] Abhiman S., Iyer L.M., Aravind L. (2008). BEN: a novel domain in chromatin factors and DNA viral proteins. Bioinformatics.

[31] Dai Q., Ren A., Westholm J.O., Serganov A.A., Patel D.J., Lai E.C. (2013). The BEN domain is a novel sequence-specific DNA-binding domain conserved in neural transcriptional repressors. Genes Dev..

[32] Ma L., Xie D., Luo M., Lin X., Nie H., Chen J., Gao C., Duo S., Han C. (2022). Identification and characterization of BEND2 as a key regulator of meiosis during mouse spermatogenesis. Sci. Adv..

[33] Yoshida A., Satomi K., Kobayashi E., Ryo E., Matsushita Y., Narita Y., Ichimura K., Kawai A., Mori T. (2022). Soft-tissue sarcoma with MN1-BEND2 fusion: A case report and comparison with astroblastoma. Genes Chromosomes Cancer.

[34] Palsgrove D.N., Manucha V., Park J.Y., Bishop J.A. (2023). A Low-grade Sinonasal Sarcoma Harboring EWSR1::BEND2: Expanding the Differential Diagnosis of Sinonasal Spindle Cell Neoplasms. Head Neck Pathol..

[35] Dashti N.K., Matcuk G., Agaimy A., Saoud C., Antonescu C.R. (2024). Malignant Bone-Forming Neoplasm With NIPBL::BEND2 Fusion. Genes Chromosomes Cancer.

[36] Huang M., Gong G., Deng Y., Long X., Long W., Liu Q., Zhao W., Chen R. (2023). Crosstalk between cancer cells and the nervous system. Medicine Advances.

[37] Klieser E., Urbas R., Stättner S., Primavesi F., Jäger T., Dinnewitzer A., Mayr C., Kiesslich T., Holzmann K., Di Fazio P. (2017). Comprehensive immunohistochemical analysis of histone deacetylases in pancreatic neuroendocrine tumors: HDAC5 as a predictor of poor clinical outcome. Hum. Pathol..

[38] Schmitz R.L., Weissbach J., Kleilein J., Bell J., Hüttelmaier S., Viol F., Clauditz T., Grabowski P., Laumen H., Rosendahl J. (2021). Targeting HDACs in Pancreatic Neuroendocrine Tumor Models. Cells.

[39] Jin N., Lubner S.J., Mulkerin D.L., Rajguru S., Carmichael L., Chen H., Holen K.D., LoConte N.K. (2016). A Phase II Trial of a Histone Deacetylase Inhibitor Panobinostat in Patients With Low-Grade Neuroendocrine Tumors. Oncologist.

[40] Kim J.Y., Kim M.S., Kim K.S., Song K.B., Lee S.H., Hwang D.W., Kim K.P., Kim H.J., Yu E., Kim S.C. (2015). Clinicopathologic and prognostic significance of multiple hormone expression in pancreatic neuroendocrine tumors. Am. J. Surg. Pathol..

[41] Byrnes L.E., Wong D.M., Subramaniam M., Meyer N.P., Gilchrist C.L., Knox S.M., Tward A.D., Ye C.J., Sneddon J.B. (2018). Lineage dynamics of murine pancreatic development at single-cell resolution. Nat. Commun..

[42] Szlachcic W.J., Ziojla N., Kizewska D.K., Kempa M., Borowiak M. (2021). Endocrine Pancreas Development and Dysfunction Through the Lens of Single-Cell RNA-Sequencing. Front. Cell Dev. Biol..

[43] Augsornworawat P., Maxwell K.G., Velazco-Cruz L., Millman J.R. (2021). Single-cell transcriptome profiling reveals beta cell maturation in stem cell-derived islets after transplantation. Cell Rep..

[44] Cherchir F., Essayeh S., Mekni S., Ben Hilel W., Gargouri F., Khiari K., Ben Nacef I., Rojbi I. (2023). Paraneoplastic Cushing syndrome caused by a pancreatic neuroendocrine tumor: a case report. Annals of Pancreatic Cancer.

[45] Kaltsas G., Androulakis I.I., de Herder W.W., Grossman A.B. (2010). Paraneoplastic syndromes secondary to neuroendocrine tumours. Endocr. Relat. Cancer.

[46] Cancer Genome Atlas Research Network Electronic address andrew_aguirre@dfci harvard edu, Cancer Genome Atlas Research Network (2017). Integrated Genomic Characterization of Pancreatic Ductal Adenocarcinoma. Cancer Cell.

[47] Gao J., Aksoy B.A., Dogrusoz U., Dresdner G., Gross B., Sumer S.O., Sun Y., Jacobsen A., Sinha R., Larsson E. (2013). Integrative analysis of complex cancer genomics and clinical profiles using the cBioPortal. Sci. Signal..

[48] Chen S. (2023). Ultrafast one-pass FASTQ data preprocessing, quality control, and deduplication using fastp. iMeta.

[49] Dobin A., Davis C.A., Schlesinger F., Drenkow J., Zaleski C., Jha S., Batut P., Chaisson M., Gingeras T.R. (2013). STAR: ultrafast universal RNA-seq aligner. Bioinformatics.

[50] Liao Y., Smyth G.K., Shi W. (2014). featureCounts: an efficient general purpose program for assigning sequence reads to genomic features. Bioinformatics.

[51] Martin M. (2011). Cutadapt removes adapter sequences from high-throughput sequencing reads. EMBnet J..

[52] Haas B.J., Dobin A., Stransky N., Li B., Yang X., Tickle T., Bankapur A., Ganote C., Doak T.G., Pochet N. (2017). STAR-Fusion: Fast and Accurate Fusion Transcript Detection from RNA-Seq. bioRxiv.

[53] Su X., Malouf G. (2023). P482: ACCUFUSION: A HIGHLY SCALABLE SOFTWARE TOOL FOR DETECTING GENE FUSIONS BY RNA-SEQ IN LEUKEMIA. HemaSphere.

[54] Becht E., Giraldo N.A., Lacroix L., Buttard B., Elarouci N., Petitprez F., Selves J., Laurent-Puig P., Sautès-Fridman C., Fridman W.H., de Reyniès A. (2016). Estimating the population abundance of tissue-infiltrating immune and stromal cell populations using gene expression. Genome Biol..

[55] Yoshihara K., Shahmoradgoli M., Martínez E., Vegesna R., Kim H., Torres-Garcia W., Treviño V., Shen H., Laird P.W., Levine D.A. (2013). Inferring tumour purity and stromal and immune cell admixture from expression data. Nat. Commun..

[56] Barbie D.A., Tamayo P., Boehm J.S., Kim S.Y., Moody S.E., Dunn I.F., Schinzel A.C., Sandy P., Meylan E., Scholl C. (2009). Systematic RNA interference reveals that oncogenic KRAS-driven cancers require TBK1. Nature.

[57] Ritchie M.E., Phipson B., Wu D., Hu Y., Law C.W., Shi W., Smyth G.K. (2015). limma powers differential expression analyses for RNA-sequencing and microarray studies. Nucleic Acids Res..

[58] Lu X., Meng J., Zhou Y., Jiang L., Yan F. (2021). MOVICS: an R package for multi-omics integration and visualization in cancer subtyping. Bioinformatics.

[59] Wu T., Hu E., Xu S., Chen M., Guo P., Dai Z., Feng T., Zhou L., Tang W., Zhan L. (2021). clusterProfiler 4.0: A universal enrichment tool for interpreting omics data. Innovation.

[60] Zhang J., Li H., Tao W., Zhou J. (2025). GseaVis: An R Package for Enhanced Visualization of Gene Set Enrichment Analysis in Biomedicine. Media Res..

[61] Hoshida Y. (2010). Nearest template prediction: a single-sample-based flexible class prediction with confidence assessment. PLoS One.

[62] Geeleher P., Cox N., Huang R.S. (2014). pRRophetic: an R package for prediction of clinical chemotherapeutic response from tumor gene expression levels. PLoS One.

[63] Fleming S.J., Chaffin M.D., Arduini A., Akkad A.D., Banks E., Marioni J.C., Philippakis A.A., Ellinor P.T., Babadi M. (2023). Unsupervised removal of systematic background noise from droplet-based single-cell experiments using CellBender. Nat Methods.

[64] Wolock S.L., Lopez R., Klein A.M. (2019). Scrublet: Computational Identification of Cell Doublets in Single-Cell Transcriptomic Data. Cell Syst..

[65] Hao Y., Stuart T., Kowalski M.H., Choudhary S., Hoffman P., Hartman A., Srivastava A., Molla G., Madad S., Fernandez-Granda C., Satija R. (2024). Dictionary learning for integrative, multimodal and scalable single-cell analysis. Nat. Biotechnol..

[66] Choudhary S., Satija R. (2022). Comparison and evaluation of statistical error models for scRNA-seq. Genome Biol..

[67] Aran D., Looney A.P., Liu L., Wu E., Fong V., Hsu A., Chak S., Naikawadi R.P., Wolters P.J., Abate A.R. (2019). Reference-based analysis of lung single-cell sequencing reveals a transitional profibrotic macrophage. Nat. Immunol..

[68] Aibar S., González-Blas C.B., Moerman T., Huynh-Thu V.A., Imrichova H., Hulselmans G., Rambow F., Marine J.-C., Geurts P., Aerts J. (2017). SCENIC: single-cell regulatory network inference and clustering. Nat. Methods.

[69] Jin S., Guerrero-Juarez C.F., Zhang L., Chang I., Ramos R., Kuan C.-H., Myung P., Plikus M.V., Nie Q. (2021). Inference and analysis of cell-cell communication using CellChat. Nat. Commun..

[70] Shannon P., Markiel A., Ozier O., Baliga N.S., Wang J.T., Ramage D., Amin N., Schwikowski B., Ideker T. (2003). Cytoscape: a software environment for integrated models of biomolecular interaction networks. Genome Res..

[71] Subramanian A., Narayan R., Corsello S.M., Peck D.D., Natoli T.E., Lu X., Gould J., Davis J.F., Tubelli A.A., Asiedu J.K. (2017). A Next Generation Connectivity Map: L1000 Platform and the First 1,000,000 Profiles. Cell.

[72] Dyer S.C., Austine-Orimoloye O., Azov A.G., Barba M., Barnes I., Barrera-Enriquez V.P., Becker A., Bennett R., Beracochea M., Berry A. (2025). Ensembl 2025. Nucleic Acids Res..

[73] Liberzon A., Subramanian A., Pinchback R., Thorvaldsdóttir H., Tamayo P., Mesirov J.P. (2011). Molecular signatures database (MSigDB) 3.0. Bioinformatics.

[74] Franzén O., Gan L.M., Björkegren J.L.M. (2019). PanglaoDB: a web server for exploration of mouse and human single-cell RNA sequencing data. Database.

[75] Yang W., Soares J., Greninger P., Edelman E.J., Lightfoot H., Forbes S., Bindal N., Beare D., Smith J.A., Thompson I.R. (2013). Genomics of Drug Sensitivity in Cancer (GDSC): a resource for therapeutic biomarker discovery in cancer cells. Nucleic Acids Res..

[76] Lu X., Li K., Li Z., Lin A., Zhao L., Shen R., Xu Z., Gao J., Lv D., Zhang Y. (2025). FigureYa: A Standardized Visualization Framework for Enhancing Biomedical Data Interpretation and Research Efficiency. iMetaMed.

[77] Peran I., Madhavan S., Byers S.W., McCoy M.D. (2018). Curation of the Pancreatic Ductal Adenocarcinoma Subset of the Cancer Genome Atlas Is Essential for Accurate Conclusions about Survival-Related Molecular Mechanisms. Clin. Cancer Res..

[78] Delpero J.R., Bachellier P., Regenet N., Le Treut Y.P., Paye F., Carrere N., Sauvanet A., Autret A., Turrini O., Monges-Ranchin G., Boher J.M. (2014). Pancreaticoduodenectomy for pancreatic ductal adenocarcinoma: a French multicentre prospective evaluation of resection margins in 150 evaluable specimens. HPB.

[79] Verbeke C.S., Gladhaug I.P. (2012). Resection margin involvement and tumour origin in pancreatic head cancer. Br. J. Surg..

[80] Méchine-Neuville A., Chenard M.P., Gairard B., Mathelin C., Bellocq J.P. (2000). [Large sections in routine breast pathology. A technique adapted to conservative surgery]. Ann. Pathol..

[81] Leek J.T., Johnson W.E., Parker H.S., Jaffe A.E., Storey J.D. (2012). The sva package for removing batch effects and other unwanted variation in high-throughput experiments. Bioinformatics.

[82] Hafemeister C., Satija R. (2019). Normalization and variance stabilization of single-cell RNA-seq data using regularized negative binomial regression. Genome Biol..

[83] Baron M., Veres A., Wolock S.L., Faust A.L., Gaujoux R., Vetere A., Ryu J.H., Wagner B.K., Shen-Orr S.S., Klein A.M. (2016). A Single-Cell Transcriptomic Map of the Human and Mouse Pancreas Reveals Inter- and Intra-cell Population Structure. Cell Syst..

[84] Tsoucas D., Dong R., Chen H., Zhu Q., Guo G., Yuan G.C. (2019). Accurate estimation of cell-type composition from gene expression data. Nat. Commun..

[85] Lu X., Meng J., Wang H., Zhou Y., Zhou J., Ruan X., Chen Y., Ye Y., Su L., Fan X. (2023). DNA replication stress stratifies prognosis and enables exploitable therapeutic vulnerabilities of HBV-associated hepatocellular carcinoma: An *in-silico* precision oncology strategy. Innov. Med..

[86] Su X., Lu X., Bazai S.K., Dainese L., Verschuur A., Dumont B., Mouawad R., Xu L., Cheng W., Yan F. (2023). Delineating the interplay between oncogenic pathways and immunity in anaplastic Wilms tumors. Nat. Commun..

[87] Wilkerson M.D., Hayes D.N. (2010). ConsensusClusterPlus: a class discovery tool with confidence assessments and item tracking. Bioinformatics.

[88] John C.R., Watson D., Russ D., Goldmann K., Ehrenstein M., Pitzalis C., Lewis M., Barnes M. (2020). M3C: Monte Carlo reference-based consensus clustering. Sci. Rep..

[89] Zhu J., Zhu Y., Wang X., Cheng W., Wang S., Yang J., Wang W., Wang Y., Meng J., Lu X., Yan F. (2024). MOVICShiny: An interactive website for multi-omics integration and visualisation in cancer subtyping. Clin. Transl. Med..

[90] Lu X., Jiang L., Zhang L., Zhu Y., Hu W., Wang J., Ruan X., Xu Z., Meng X., Gao J. (2019). Immune Signature-Based Subtypes of Cervical Squamous Cell Carcinoma Tightly Associated with Human Papillomavirus Type 16 Expression, Molecular Features, and Clinical Outcome. Neoplasia.

[91] Muzny D.M., Bainbridge M.N., Chang K., Dinh H.H., Drummond J.A., Fowler G., Kovar C.L., Lewis L.R., Morgan M.B., Newsham I.F. (2012). Comprehensive molecular characterization of human colon and rectal cancer. Nature.

