# Supplementary material for: Molecular taxonomy of pancreatic neuroendocrine tumors reveals BEND2-fusions-driven transcriptional plasticity and therapeutic vulnerabilities
Source: Cell Rep Med. 2026 Mar 6;7(3):102642. doi: 10.1016/j.xcrm.2026.102642 (PMC13006400; doi:10.1016/j.xcrm.2026.102642)
Supplement: Document S1. Figures S1–S17 [file mmc1.pdf]

Cell Reports Medicine, Volume *7*

Supplemental information

Molecular taxonomy of pancreatic neuroendocrine tumors reveals *BEND2*-fusions-driven transcriptional plasticity and therapeutic vulnerabilities

Xiaofan Lu, Philippe Baltzinger, Li Xu, Antonin Fattori, Sehrish Khan Bazai, Fatima Alhourani, Marie-Pierrette Chenard, Philippe Bachellier, Pietro Addeo, Véronique Debien, Clara Vacheret, Alessio Imperiale, Patrick Pessaux, Wenxuan Cheng, Martin Balzinger, Jean-Emmanuel Kurtz, Irwin Davidson, Xiaoping Su, Bernard Goichot, and Gabriel G. Malouf

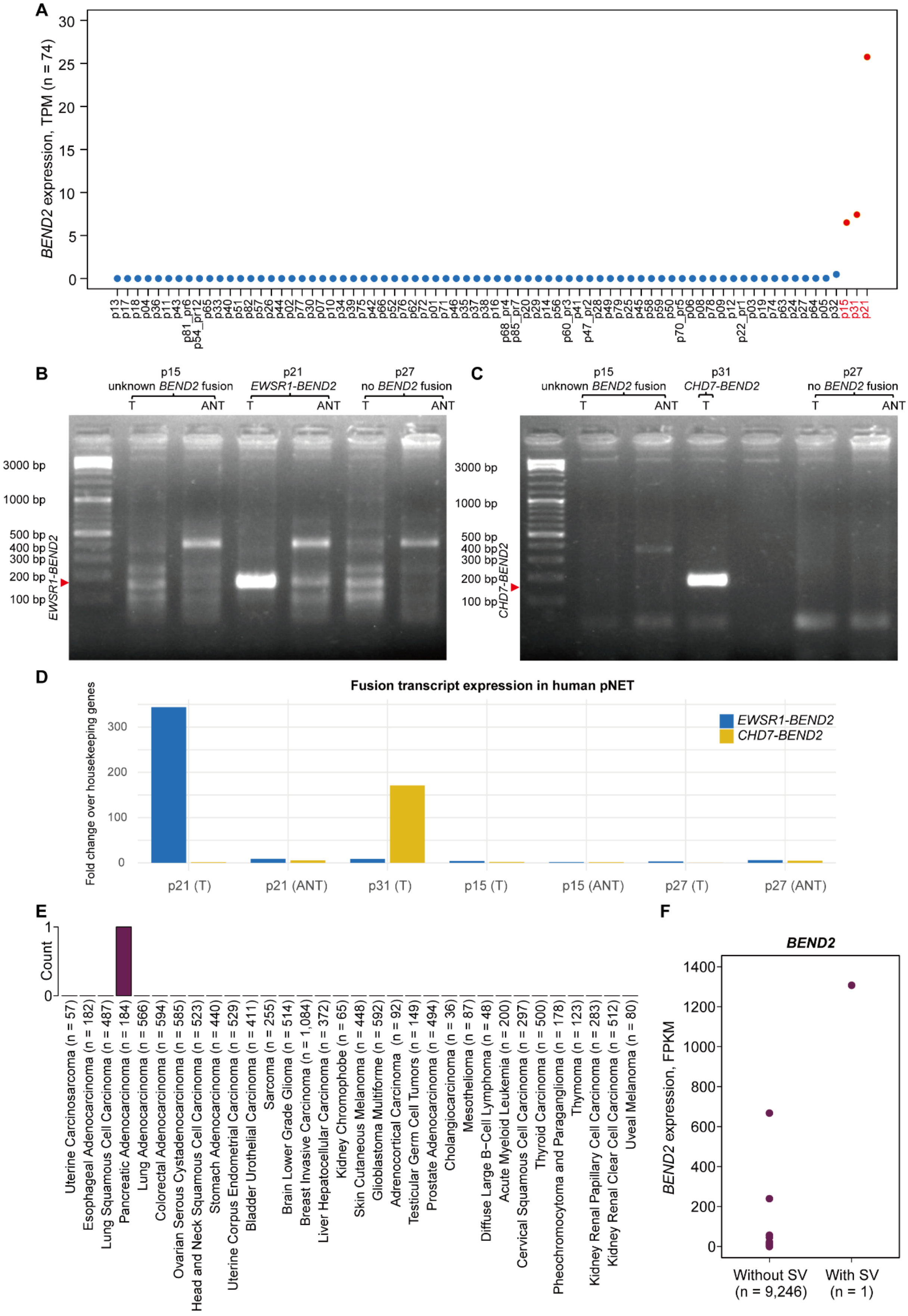

Figure S1. ***BEND2*** **expression and rearrangement in the current and TCGA cohorts. Related to Figure 1.** (**A**) *BEND2* expression across all samples in the current study; samples with identified *BEND2* fusions are

highlighted in red.

(**B**) RT-PCR validation of *EWSR1–BEND2* fusion transcript in tumor (T) and adjacent non-tumor (ANT) samples from p15 (unknown *BEND2* fusion), p21 (*EWSR1–BEND2* fusion), and p27 (no *BEND2* fusion).

(**C**) RT-PCR validation of *CHD7–BEND2* fusion transcript in tumor and adjacent non-tumor samples from p15 (unknown *BEND2* fusion), p31 (*CHD7–BEND2* fusion), and p27 (no *BEND2* fusion).

(**D**) Quantitative PCR (qPCR) analysis of fusion transcript expression relative to housekeeping genes.

(**E**) Frequency of *BEND2* rearrangements across cancer types in 10,967 TCGA cases.

(**F**) *BEND2* expression levels in 9,279 TCGA samples with available RNA-seq data.

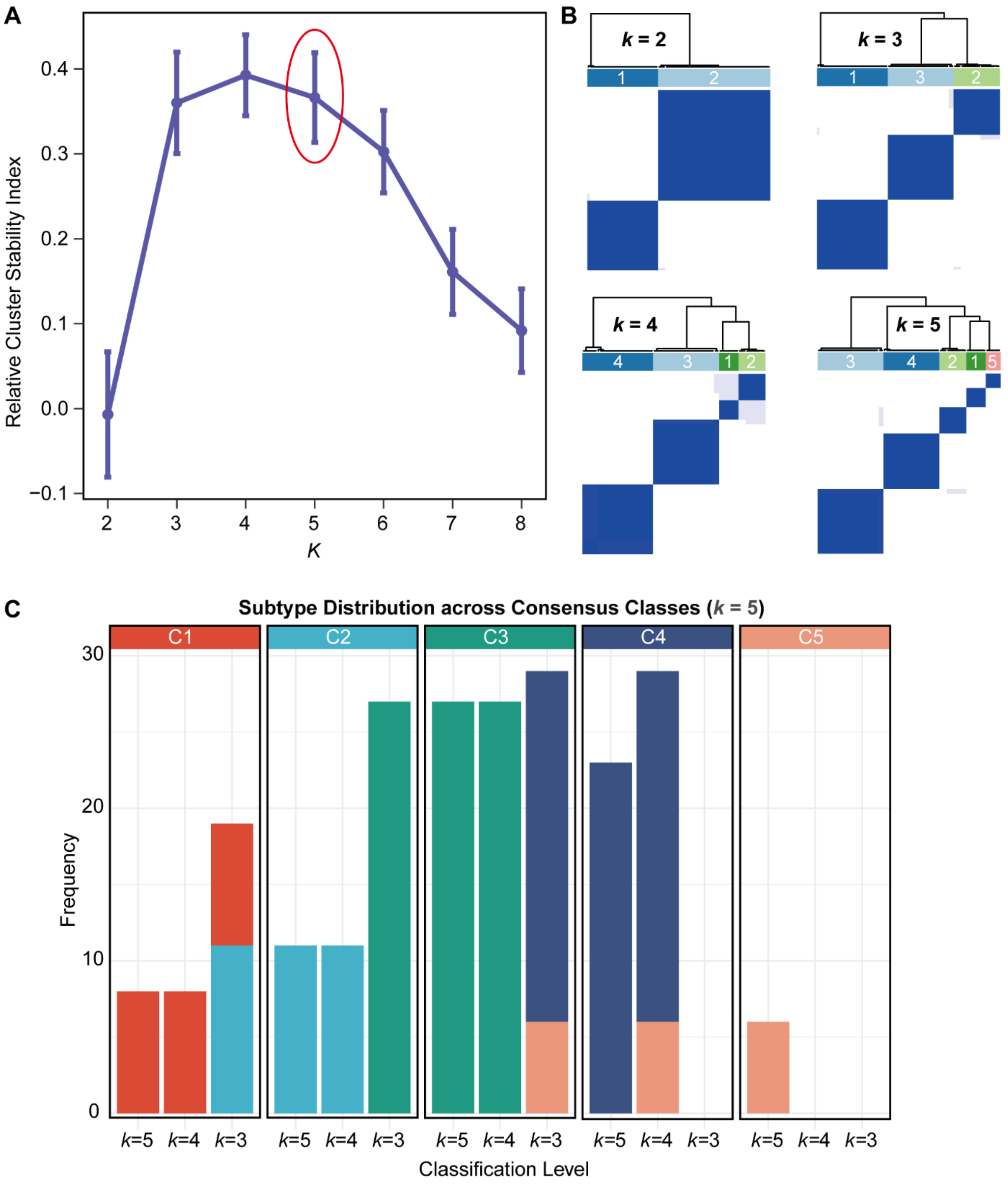

Figure S2. Identification of optimal clustering number. Related to Figure 3.

(**A**) Line chart showing stability index of Monte Carlo reference-based consensus clustering (clustering number *k* from 2 to 8) on transcriptome expression of 75 primary pNETs in our cohort; High stability maintains at *k* = 5 and maximizes inter-tumor heterogeneity.

(**B**) Consensus heatmap with clustering number *k* from 2 to 5 demonstrated the clearest boundary when *k* = 5.

(**C**) Correspondence of sample classifications across clustering resolutions (*k* = 3, 4, and 5). Each barplot shows how samples assigned to a given cluster at one resolution map to clusters at the next, illustrating how groups split or merge across k.

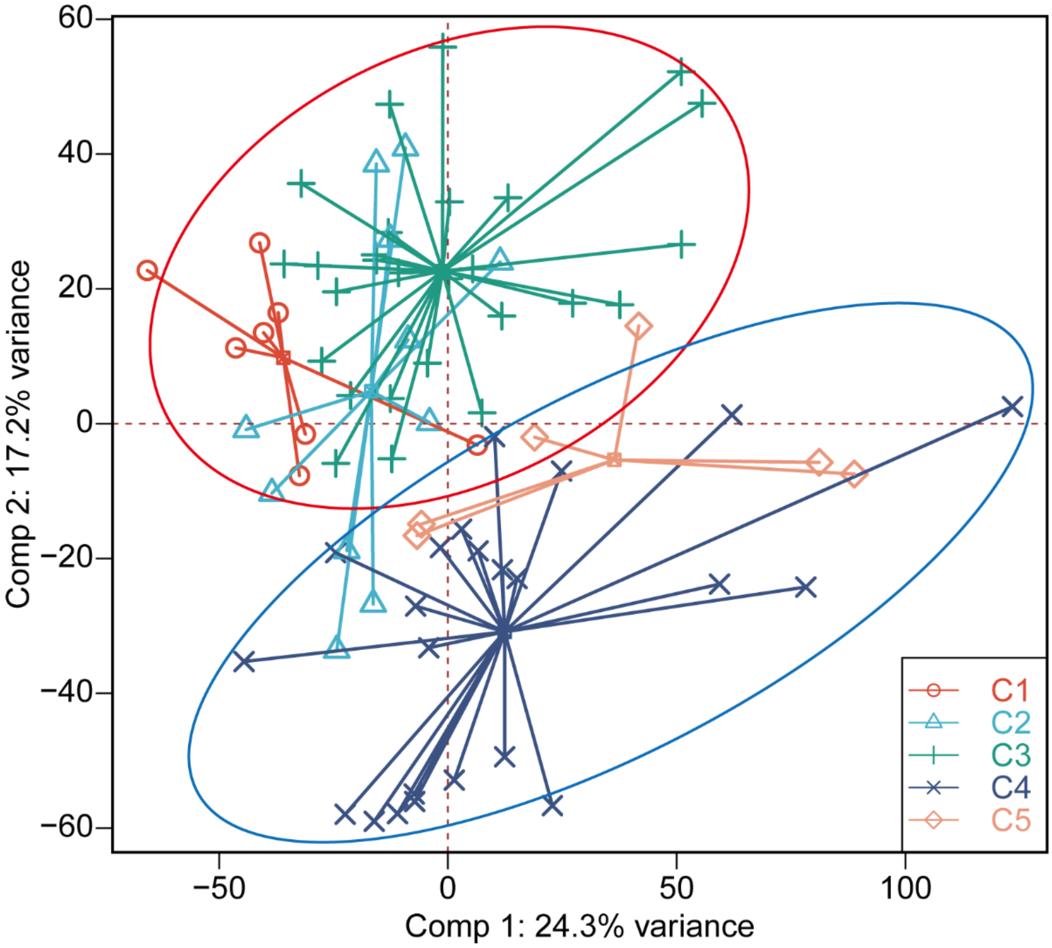

Figure S3. Inter-tumor heterogeneity. Related to Figure 3.

Principal component analysis using mRNA expression confirmed the molecular distinctions among the four subgroups; two circles revealed greater potential for intra-subtype heterogeneity between C1-3 and C4-5.

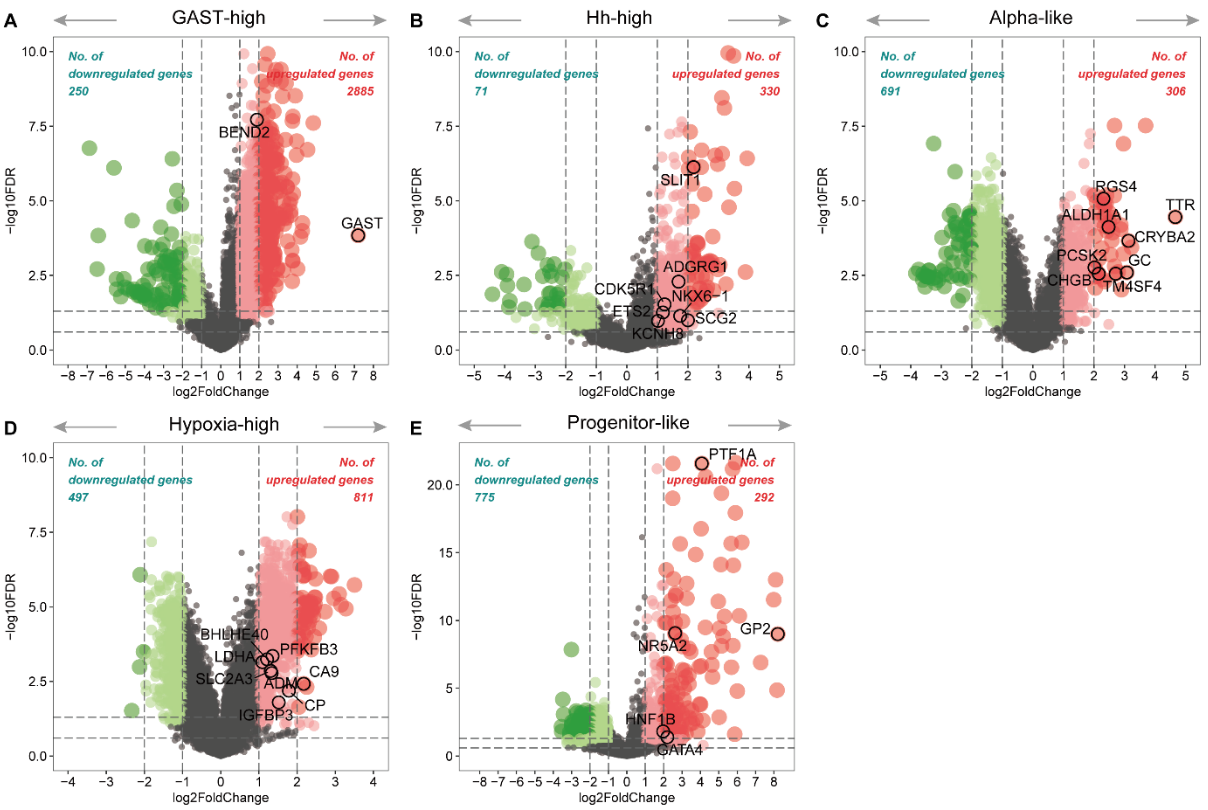

Figure S4. Differential expression and specific upregulated genes for each bulk subtype. Related to Figure 3.

(**A**) Volcano plot showing differentially expressed genes between GAST-high subtype versus others. The number of dysregulated genes was identified using the threshold of $\log_2 FC = 1$ and FDR < 0.05.

(**B**) Same as (**A**) but for Hh-high subtype.

(**C**) Same as (**A**) but for Alpha-like subtype.

(**D**) Same as (**A**) but for Hypoxia-high subtype.

(**E**) Same as (**A**) but for Progenitor-like subtype.

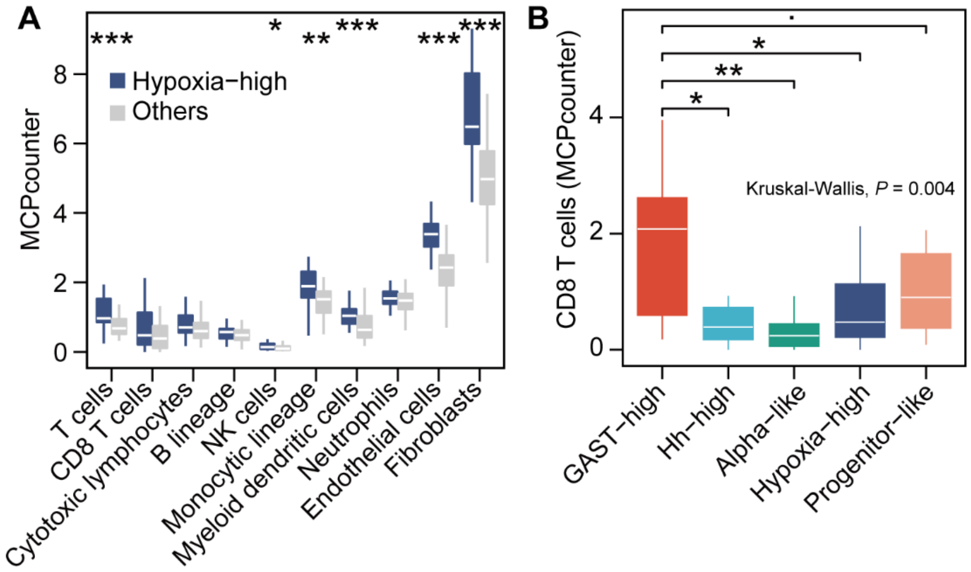

Figure S5. Distinct microenvironmental cell infiltration in Hypoxia-high and GAST-high pNET subtypes. Related to Figure 3.

(**A**) Boxplot showing the abundance of 10 microenvironmental cell types in the Hypoxia-high subtype compared to other subtypes within the pNET cohort.

(**B**) Boxplot displaying the distribution of $CD8^+$ T cell abundance, as measured by MCPcounter, across the different molecular subtypes. Overall statistical significance was assessed using the Kruskal-Wallis test, with pairwise comparisons evaluated by the Mann-Whitney U test (. $P < 0.1$, * $P < 0.05$, ** $P < 0.01$, *** $P < 0.001$).

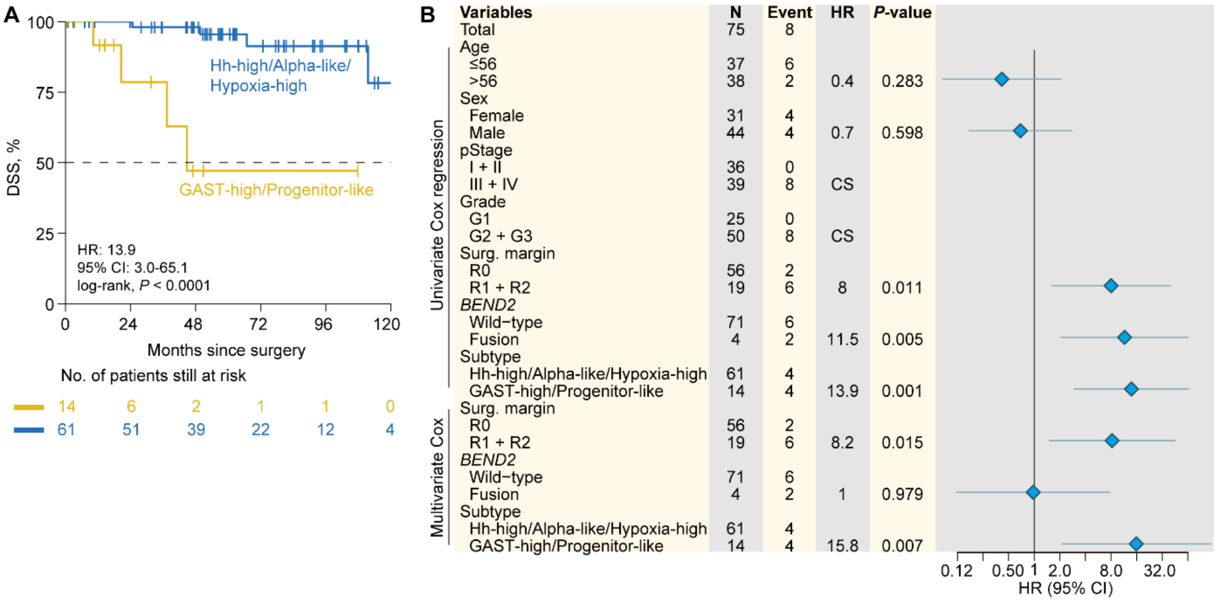

Figure S6. Prognostic relevance of transcriptomic subtypes. Related to Figure 3.

(**A**) Kaplan-Meier curves showing poor clinical outcomes between an aggressive pNET (i.e., GAST-high/Progenitor-like subtypes) and nonaggressive pNET (i.e., Hh-high/Alpha-like/Hypoxia-high subtypes) regarding disease-specific survival (DSS).

(**B**) Forest plot showing the hazard ratio (95% CI) in the univariate Cox proportional hazards regression and multivariate regression after adjusting for major clinicopathological features and the corresponding *P*-values. The complete separation (CS) indicates when the outcome variable complete separates a predictor variable, leading to an inaccurate estimation of the hazard ratio.

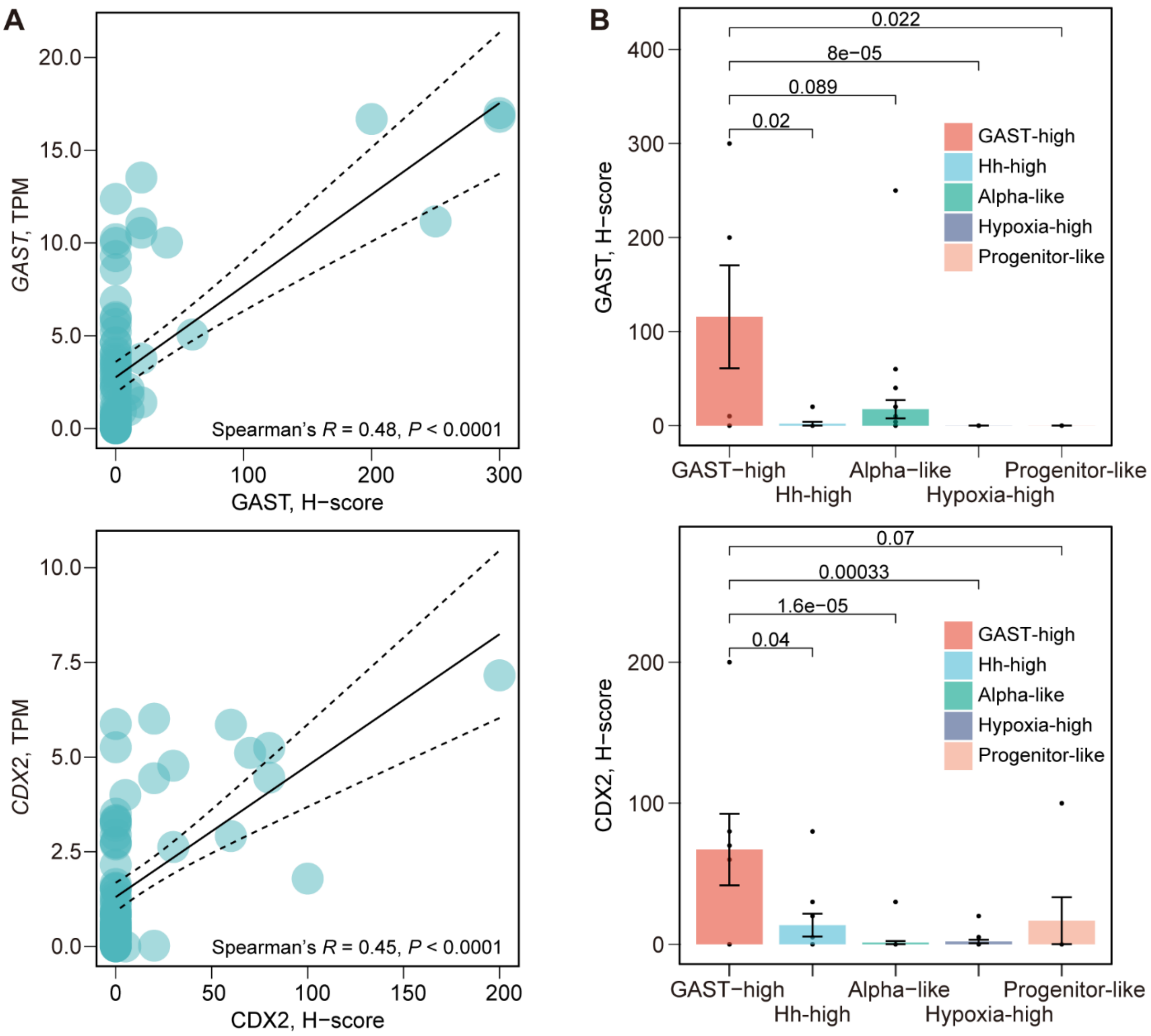

Figure S7. Immunohistochemical assessment of GAST and CDX2 in pNET subtypes. Related to Figure 3.

(**A**) Correlation plots showing concordance between RNA-seq expression and IHC H-scores for GAST (top) and CDX2 (bottom).

(**B**) Barplots of H-scores for GAST (top) and CDX2 (bottom) across molecular subtypes, demonstrating enrichment in GAST-high tumors.

Statistical significance was evaluated using Spearman correlation and the Mann–Whitney U test.

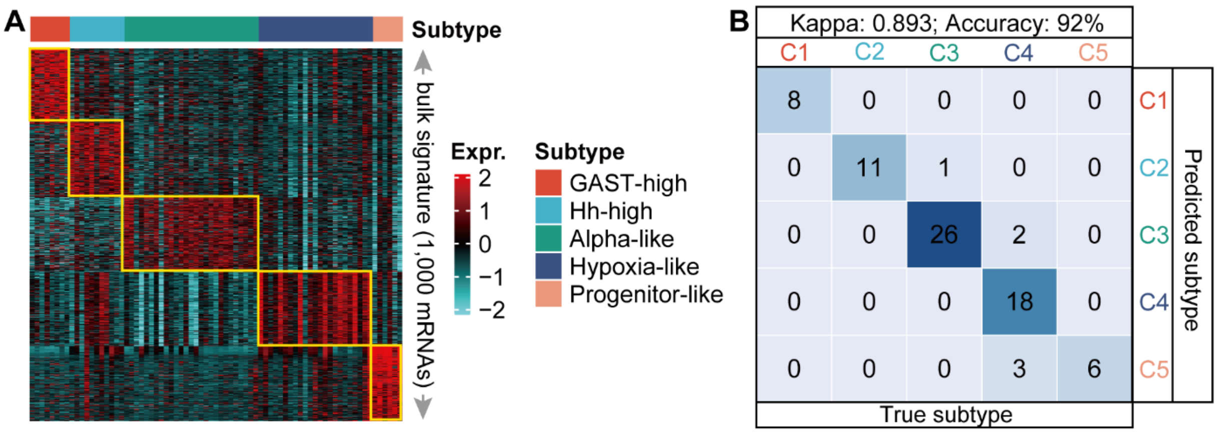

Figure S8. Identification of a 1000-gene bulk signature. Related to Figure 3.

(**A**) Heatmap showing the expression pattern of the 1000-gene signature using the top 200 uniquely and significantly upregulated mRNAs in each one of the five subtypes.

(**B**) Consistency heatmap demonstrating superior predictive performance of the 1000-gene signature deployed in our pNET cohort.

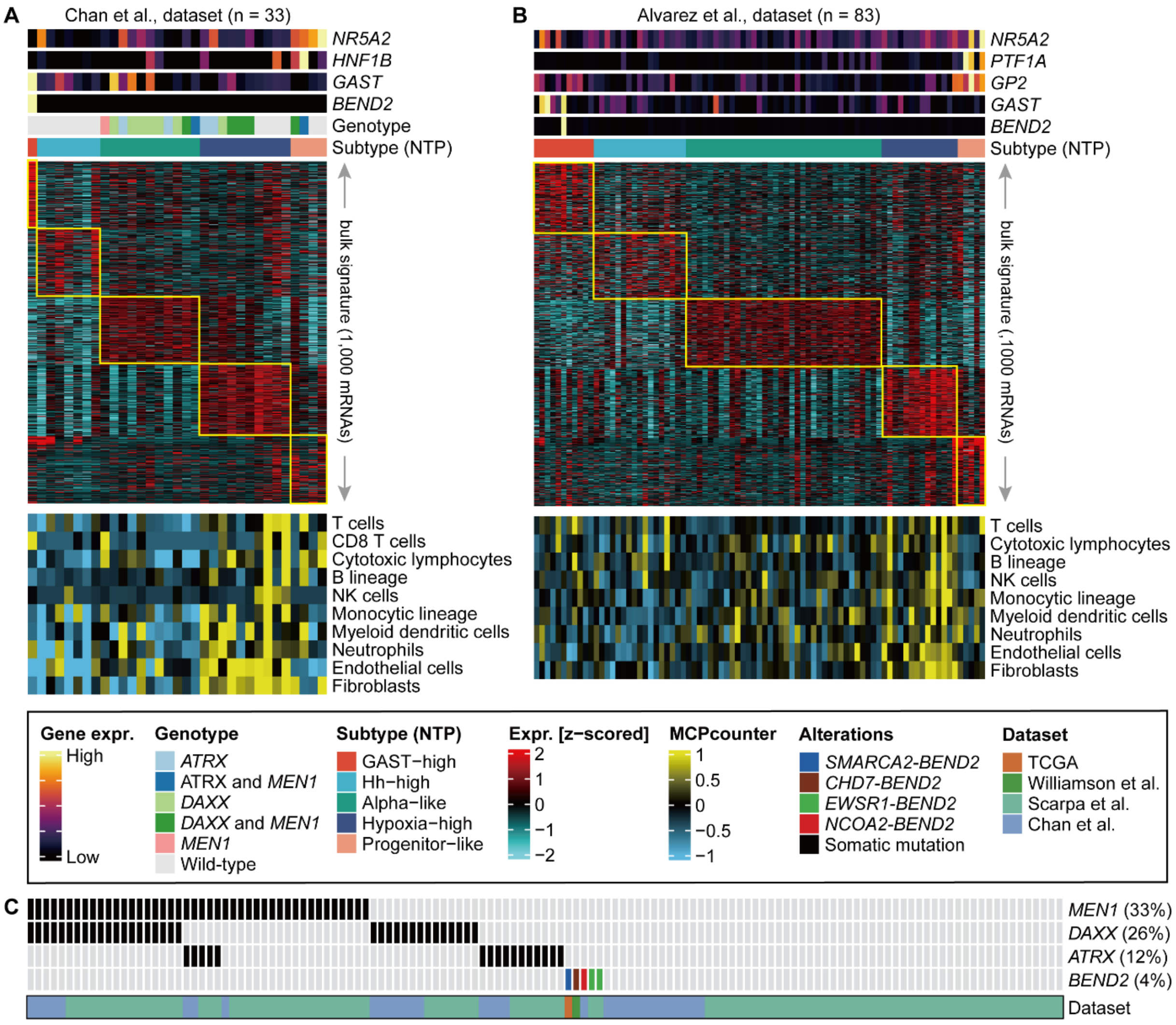

Figure S9. External validation of bulk transcriptomic subtypes and genomic context of *BEND2* fusions.

Related to Figure 3.
(**A**) Nearest template prediction reproduced five transcriptomic subtypes in Chan's cohort using the bulk signature. Heatmap showing ATRX/DAXX/MEN1 mutational status and expression of specific gene markers for aggressive subtypes (top panel), transcriptome landscape of 1000-gene bulk signature (middle panel) and tumor microenvironment profiling using MCPcounter.
(**B**) Nearest template prediction reproduced five transcriptomic subtypes in Alvarez's cohort using the bulk signature with a similar heatmap layout as Chan's cohort. Abundance of CD8 T cells was not estimated in Alvarez's cohort due to the lack of expression of *CD8B*, a specific marker for CD8 T cells in MCPcounter.
(**C**) Oncoprint summarizing genomic alterations across four independent cohorts. Each column represents an individual pNET sample. Colored bars indicate genomic events, including *BEND2* fusions and mutations in *ATRX*, *DAXX*, and *MEN1*.

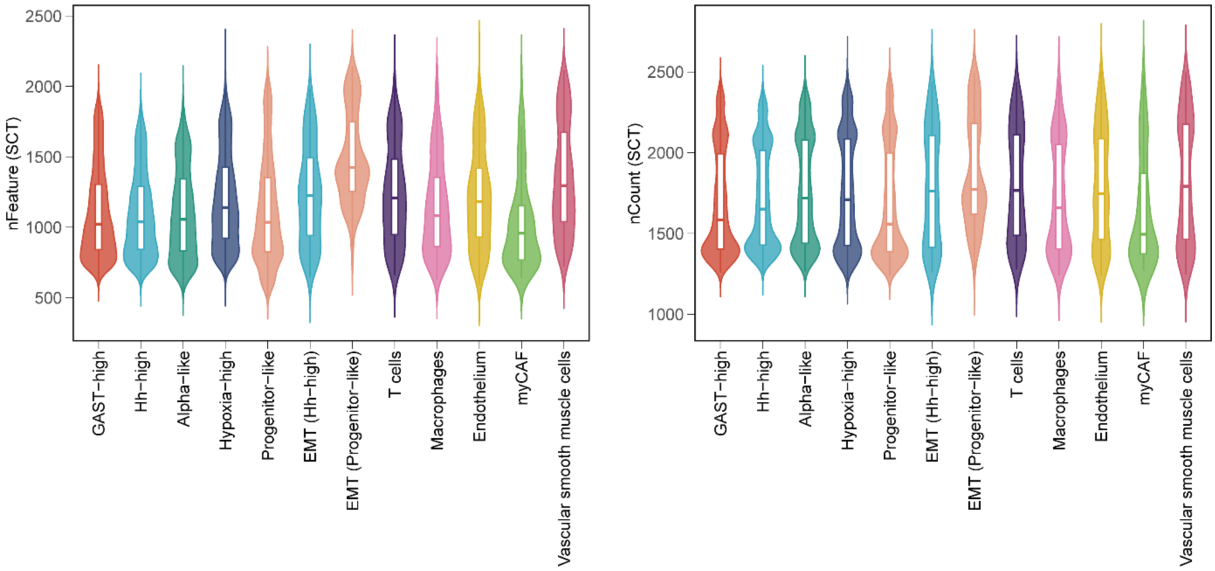

Figure S10. Quality metrics of tumor and non-tumor clusters in snRNA-seq data. Related to Figure 4. Violin plots display the distribution of (left) the number of detected genes (nFeature_SCT) and (right) unique molecular identifiers (nCount_SCT) across major tumor subtypes (GAST-high, Hh-high, Alpha-like, Hypoxia-high, Progenitor-like, and EMT-like clusters) and non-tumor cell types (T cells, macrophages, endothelium, myCAF, vascular smooth muscle cells).

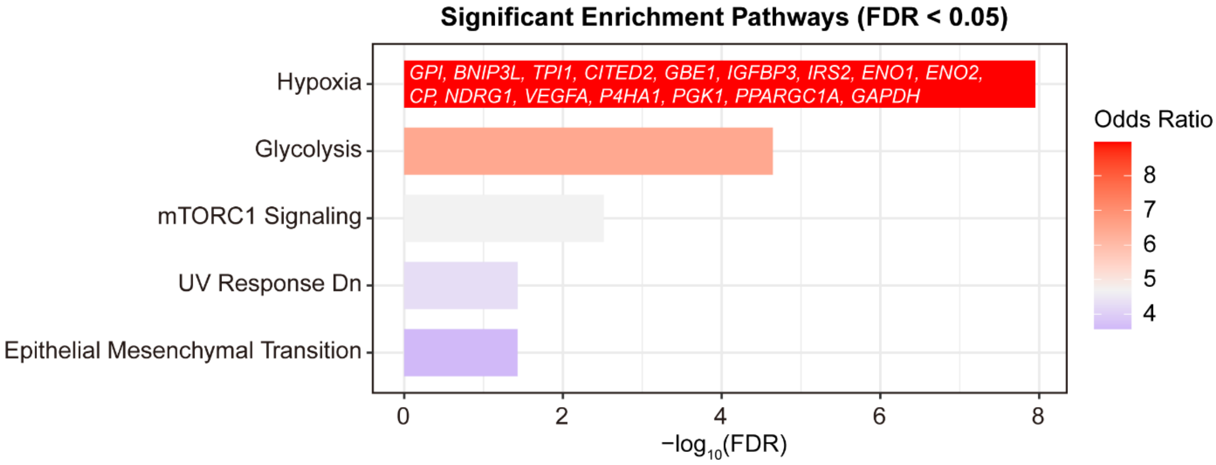

Figure S11. Pathway enrichment analysis of genes upregulated in the Hypoxia-high sn-cluster. Related to

Figure 4.
Bar lengths represent the $-\log_{10}$(FDR) values of significantly enriched Hallmark pathways (FDR < 0.05), and bar colors indicate the odds ratio. Gene members contributing to the top significant pathway (Hypoxia) are displayed within the corresponding bar.

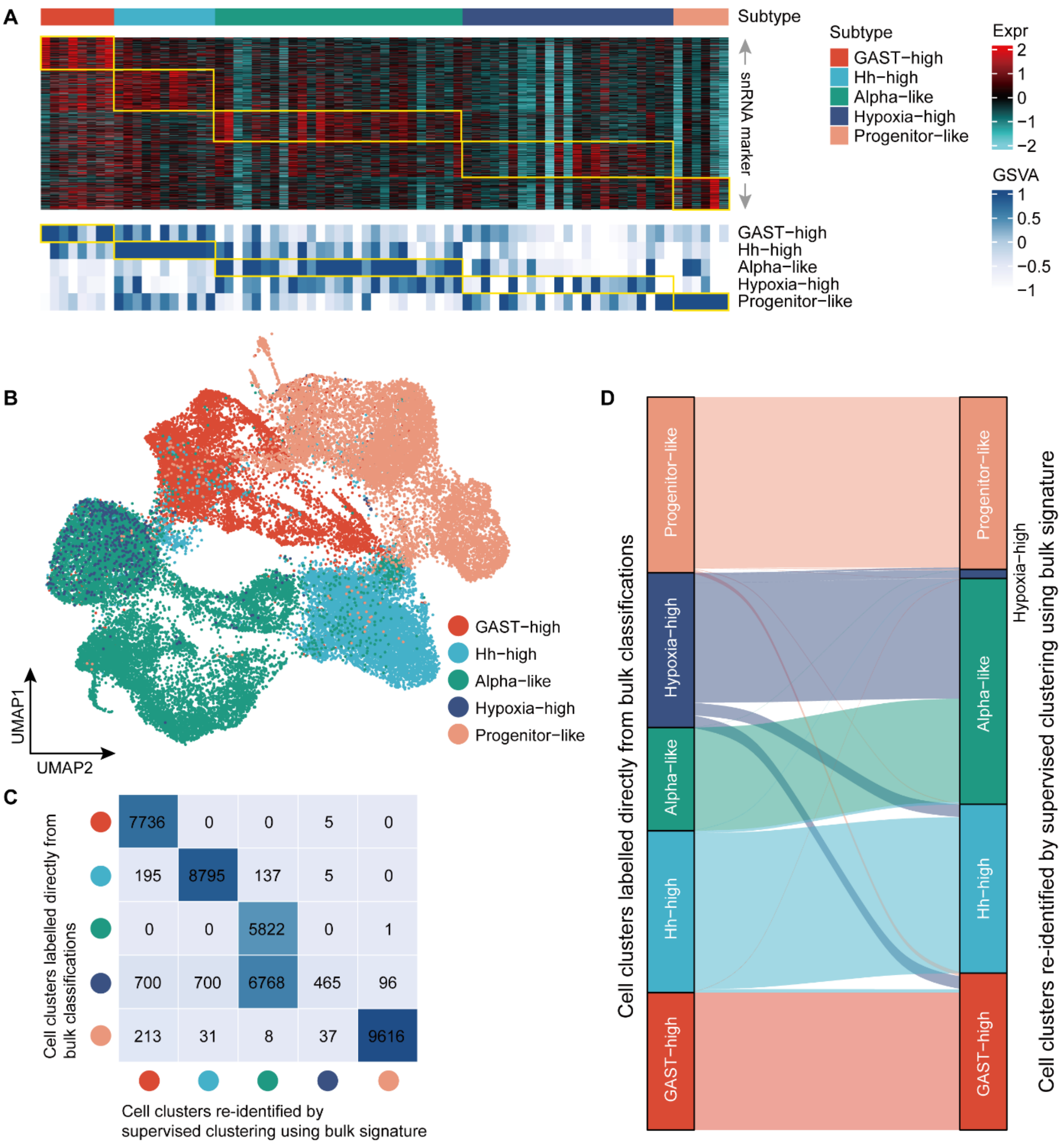

Figure S12. Cross-platform validation. Related to Figure 4.
(**A**) Heatmap showing the transcriptomic expression landscape using snRNA markers to bulk RNA-seq data across different bulk subtypes. Gene-level expression presented at the top panel while subtype-level enrichment calculated by GSVA is presented at the bottom panel.
(**B**) UMAP representation with cells classified by bulk signature.
(**C**) Contingency table between pseudo-bulk sn-clusters and re-classified cell labels using bulk signature.
(**D**) Sankey plot showing the association between pseudo-bulk sn-clusters and re-classified cell labels using bulk signature.

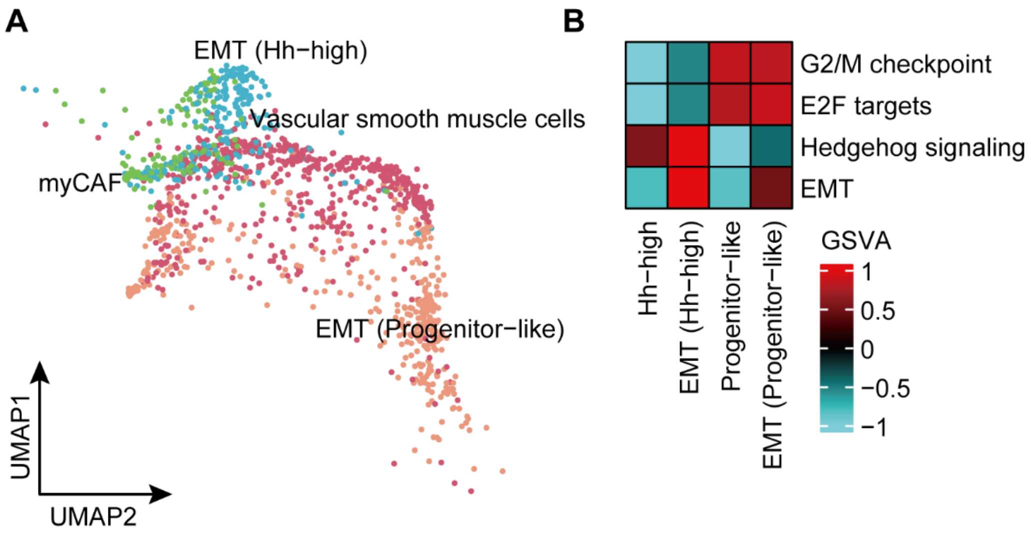

Figure S13. Sub-clustering of a mixture cell group. Related to Figure 4.

(**A**) UMAP representation with sub-clustering of a mixture cell group revealed four distinct sub-clusters, including stellate cells, vascular smooth muscle cells, and two sub-clusters of tumor cells exhibiting different EMT signatures.

(**B**) Heatmap showing that the two EMT-like clusters originated from tumor cells (one from the Hh-high sample and another one from the Progenitor-like samples) exhibit an EMT phenotype.

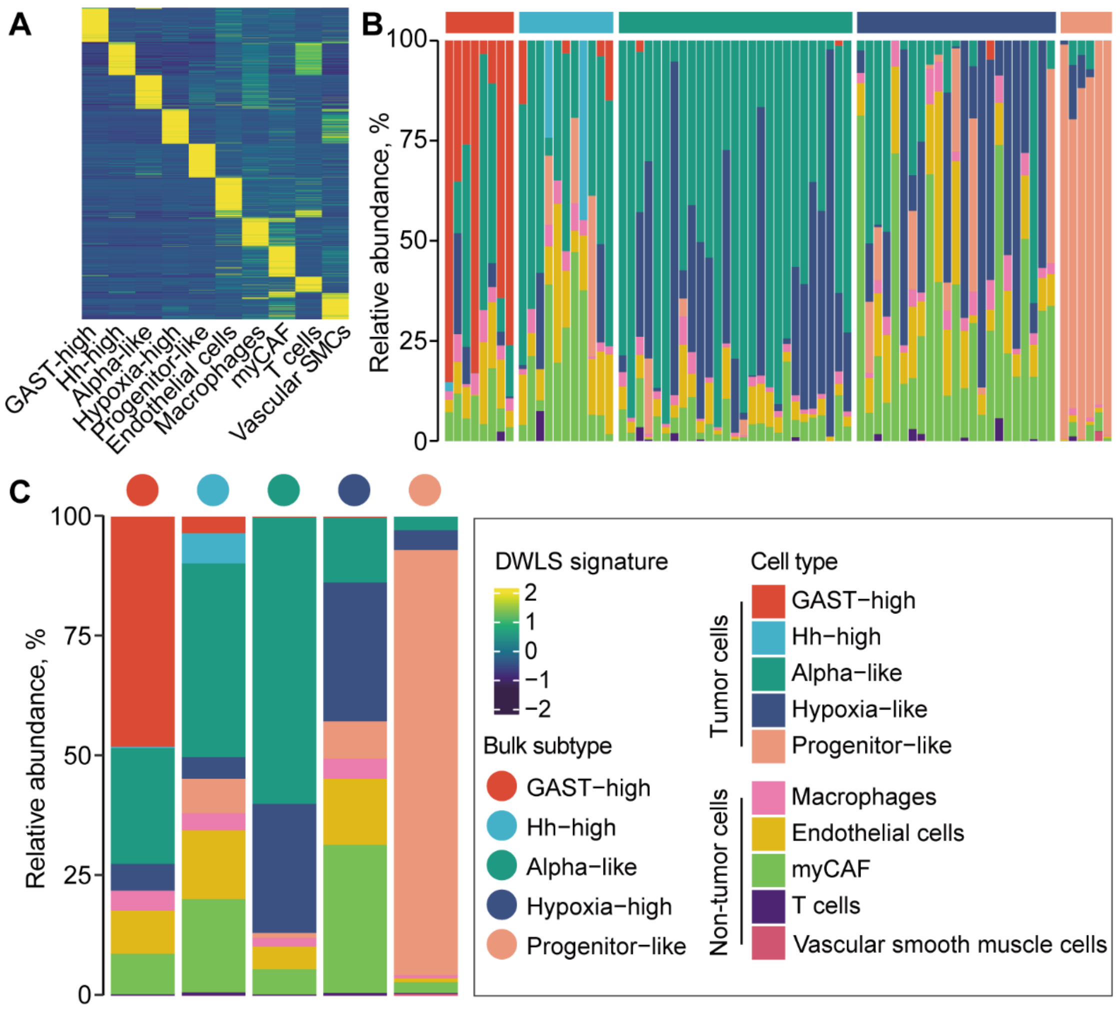

Figure S14. Deconvolution of bulk pNET transcriptomes using DWLS. Related to Figure 4.

(**A**) Heatmap of DWLS-derived signatures across the 10 snRNA-seq–defined cell populations, demonstrating clear separation of transcriptomic programs.

(**B**) Stacked bar plot showing the estimated fraction of each cell type per patient in the bulk pNET cohort.

(**C**) Stacked bar plot summarizing the subtype-level distribution of cell-type fractions across the five molecular subtypes.

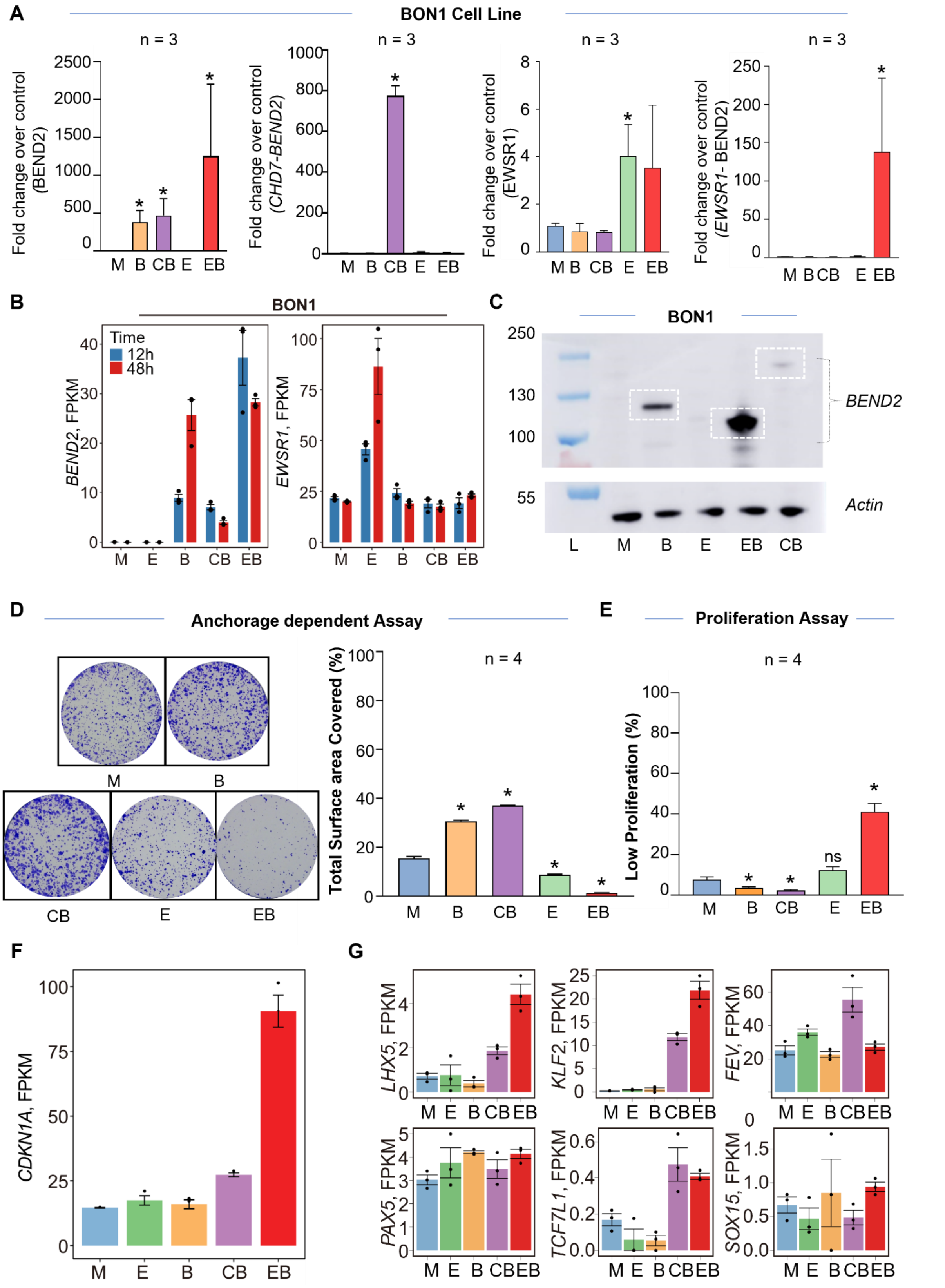

Figure S15. Functional characterization of *BEND2* fusions in BON1 cells. Related to Figure 6.

(**A**) qPCR validation of doxycycline-inducible expression in BON1 cells using primers targeting *BEND2* (B), *EWSR1* (E), and the fusion junctions for *CHD7-BEND2* (CB) and *EWSR1-BEND2* (EB). Data are represented as mean +/- SEM.

(**B**) Bar plot showing RNA-seq–based expression of transgenes at 12 and 48 hours post-induction across models.

Data are represented as mean +/- SEM.
(**C**) Western blot confirming BEND2 protein overexpression in BON1 cells. Actin served as a loading control.
(**D**) Anchorage-dependent colony formation assay. BON1 cells overexpressing B or CB formed significantly more colonies relative to controls, while E and EB overexpression reduced colony formation. Representative images are shown on the left.
(**E**) Cell proliferation assessed by CellTrace assay. BON1 cells overexpressing BEND2 (B) or *CHD7-BEND2* (CB) displayed significantly increased proliferation compared to mCherry controls (M), whereas *EWSR1* (E) and *EWSR1-BEND2* (EB) showed reduced proliferation. Data are represented as mean +/- SEM.
(**F**) Expression of *CDKN1A* across BON1 models at 48 hours, as measured by RNA-seq. Data are represented as mean +/- SEM.
(**G**) Expression of GAST-high subtype-specific regulons across BON1 models at 48 hours, as measured by RNA-seq. Data are represented as mean +/- SEM.
Data are represented as mean ± SEM from at least three independent biological replicates (each in technical triplicate). Statistical analysis was performed using GraphPad Prism 9 or R (version 4.2.2). Significance is indicated as: $P < 0.05$ (*), $P < 0.01$ (**), $P < 0.001$ (***), $P < 0.0001$ (****); ns, not significant.

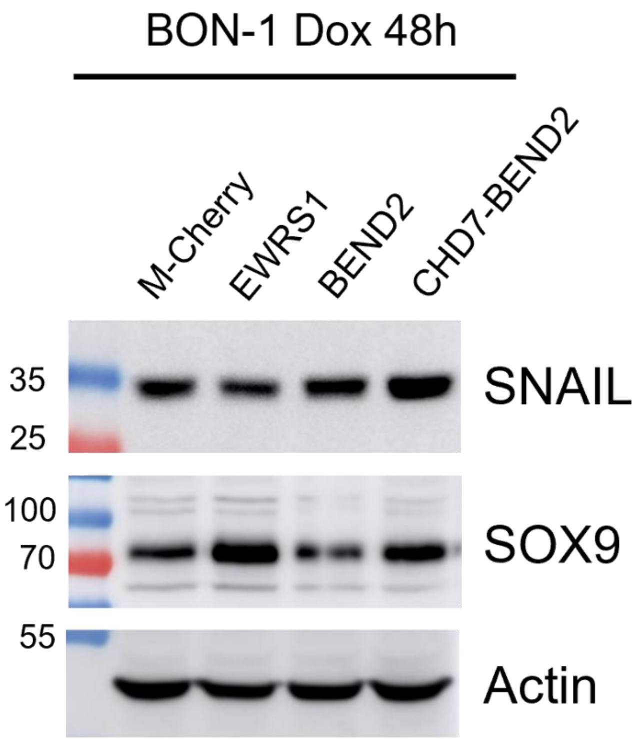

Figure S16. Western blot validation of EMT and neurodevelopmental markers in BON1 models at 48 hours. Related to Figure 6.
BON1 cells expressing mCherry (control), *EWSR1*, *BEND2*, and *CHD7-BEND2* were harvested 48 hours after doxycycline induction and analyzed by western blot. β-Actin was used as a loading control to ensure equal protein loading across lanes.

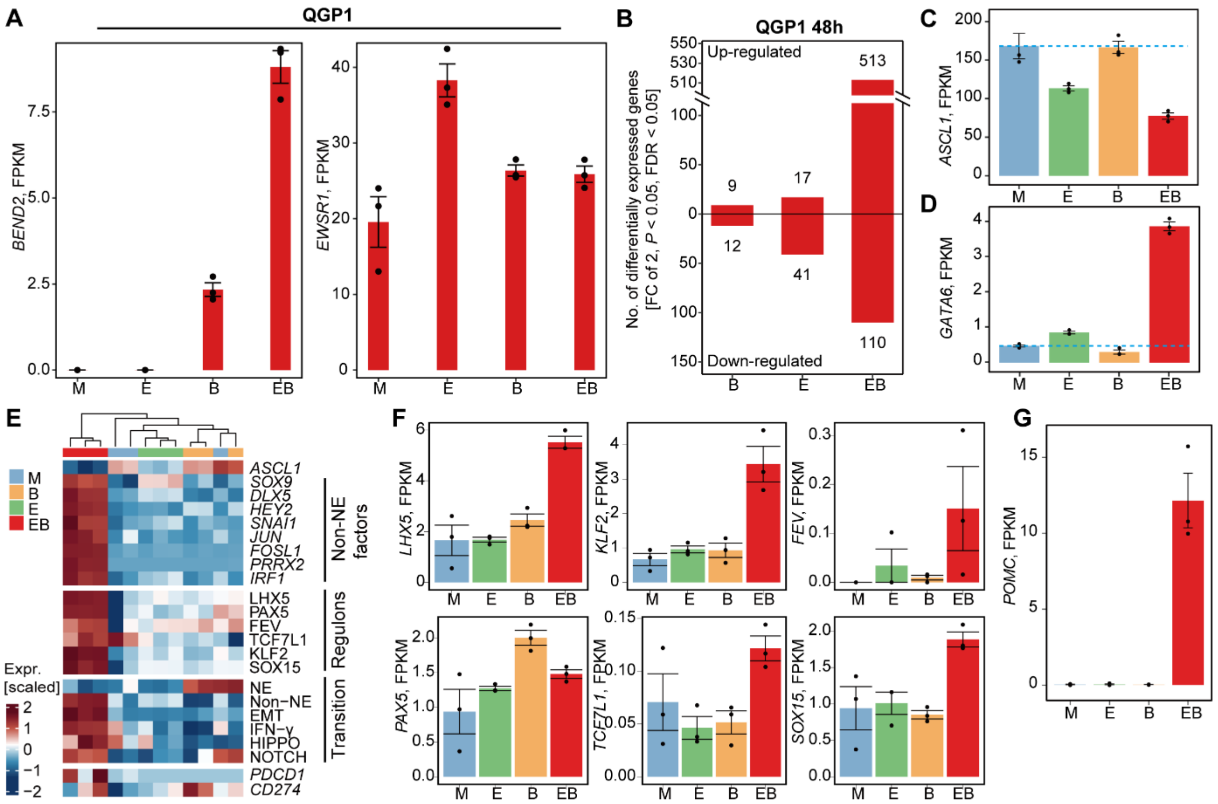

Figure S17. Validation of fusion-driven transcriptional reprogramming in QGP1 cells. Related to Figure 6.

(**A**) Bar plot showing successful overexpression of transgenes in QGP1 cells at 48 hours across different models, including *EWSR1* (E), *BEND2* (B), and *EWSR1-BEND2* (EB), compared to the mCherry (M) control. Data are represented as mean +/- SEM.

(**B**) Bar plot showing the number of differentially expressed genes in QGP1 cell line models at 48 hours.

(**C**) Bar plot of *ASCL1* expression in QGP1 models at 48 hours, showing significant downregulation in *EWSR1-BEND2* cells compared to mCherry control (FC = 0.47, $P < 0.001$, FDR = 0.014). Data are represented as mean +/- SEM.

(**D**) Bar plot showing robust upregulation of *GATA6* in QGP1 *BEND2* fusion lines at 48 hours (FC = 3.3, $P <$ 0.001, FDR < 0.001).

(**E**) Heatmap showing transcriptional activation of neurodevelopmental, mesenchymal, and immune-related TFs in *EWSR1-BEND2*-expressing QGP1 cells at 48 hours. This is accompanied by *ASCL1* downregulation, activation of GAST-high regulons, suppression of neuroendocrine (NE) signatures, and upregulation of non-NE programs and pathways including EMT (Hallmark), NOTCH signaling (GO Biological Process), HIPPO signaling (Reactome), and interferon-γ response (Hallmark), as assessed by GSVA. Expression changes in immune checkpoint genes are also shown.

(**F**) Bar plot showing expression levels of GAST-high subtype-specific regulons across QGP1 cell line models at 48 hours. Data are represented as mean +/- SEM.

(**G**) Bar plot showing marked upregulation of *POMC* in QGP1 cells expressing *EWSR1-BEND2* at 48 hours. Data are represented as mean +/- SEM.
